# Insertional mutagenesis in the zoonotic pathogen *Chlamydia caviae*

**DOI:** 10.1371/journal.pone.0224324

**Published:** 2019-11-07

**Authors:** Kimberly Filcek, Katarina Vielfort, Samada Muraleedharan, Johan Henriksson, Raphael H. Valdivia, Patrik M. Bavoil, Barbara S. Sixt

**Affiliations:** 1 Department of Microbial Pathogenesis, University of Maryland, School of Dentistry, Baltimore, MD, United States of America; 2 Laboratory for Molecular Infection Medicine Sweden (MIMS), Umeå Centre for Microbial Research, Department of Molecular Biology, Umeå University, Umeå, Sweden; 3 Department of Molecular Genetics and Microbiology, Duke University, Durham, NC, United States of America; University of Technology Sydney, AUSTRALIA

## Abstract

The ability to introduce targeted genetic modifications in microbial genomes has revolutionized our ability to study the role and mode of action of individual bacterial virulence factors. Although the fastidious lifestyle of obligate intracellular bacterial pathogens poses a technical challenge to such manipulations, the last decade has produced significant advances in our ability to conduct molecular genetic analysis in *Chlamydia trachomatis*, a major bacterial agent of infertility and blindness. Similar approaches have not been established for the closely related veterinary *Chlamydia* spp., which cause significant economic damage, as well as rare but potentially life-threatening infections in humans. Here we demonstrate the feasibility of conducting site-specific mutagenesis for disrupting virulence genes in *C*. *caviae*, an agent of guinea pig inclusion conjunctivitis that was recently identified as a zoonotic agent in cases of severe community-acquired pneumonia. Using this approach, we generated *C*. *caviae* mutants deficient for the secreted effector proteins IncA and SinC. We demonstrate that *C*. *caviae* IncA plays a role in mediating fusion of the bacteria-containing vacuoles inhabited by *C*. *caviae*. Moreover, using a chicken embryo infection model, we provide first evidence for a role of SinC in *C*. *caviae* virulence *in vivo*.

## Introduction

The bacterial genus *Chlamydia* is comprised of multiple human and animal pathogenic species that are capable of causing significant morbidity and mortality [1]. All described *Chlamydia* spp. are obligate intracellular bacteria that have a biphasic developmental cycle [2]. The infective stage, the elementary body (EB), invades the host cell in a process that leads to the formation of a pathogen-containing vacuole, named inclusion. Within this inclusion, the EB differentiates into the replicative stage, the reticulate body (RB). After several rounds of division, RBs retro-differentiate into EBs, which are released from the host cell to infect neighboring cells [3].

The main human pathogenic *Chlamydia* spp. are *Chlamydia trachomatis*, responsible for both urogenital and ocular infections [4, 5], and *Chlamydia pneumoniae*, an agent of respiratory tract infections [6]. Less prevalent, but potentially life-threatening, are zoonotic infections caused by veterinary *Chlamydia* species [7]. In this context, most frequent are infections with avian strains of *Chlamydia psittaci* [8]. While these bacteria primarily infect birds, including a wide range of wild and domesticated species, many instances of avian to human transmission have been documented [9]. The manifestation of avian chlamydiosis in humans, also known as psittacosis or ornithosis, can vary in severity from mild influenza-like illness to severe atypical pneumonia that can be fatal [7]. Zoonotic potential has also been reported for *Chlamydia abortus*, a major infectious cause of abortion in sheep, and *Chlamydia felis*, a common cause of acute and chronic conjunctivitis in cats [7]. Moreover, new isolates of *Chlamydia caviae*, a species previously restricted to cases of inclusion conjunctivitis in guinea pig pups [10], was recently identified as a zoonotic agent of severe community-acquired pneumonia in humans [11, 12]. The overall impact of animal chlamydioses on human health remains unknown, because zoonotic *Chlamydia* infections are likely underdiagnosed due to the limited awareness of physicians [7, 9].

Comparative genomic analyses have highlighted genetic differences between various representatives of human-pathogenic and veterinary *Chlamydia* species, which may in part account for the observed differences in host tropism and disease phenotypes [13–16]. For instance, while all known *Chlamydia* spp. possess a type III secretion (T3S) system [17], they encode variable sets of T3S effector proteins. We have recently described the novel T3S effector protein SinC (secreted inner nuclear membrane-associated *C**hlamydia* protein) in *C*. *psittaci* Cal-10 [18]. *C*. *psittaci* SinC displays two properties that are unprecedented for *Chlamydia* effector proteins: (1) after secretion at late stages of infection, SinC localizes to the inner nuclear membrane of the infected cell, where it associates with LEM domain proteins, including emerin and the lamin B receptor (LBR), and (2) SinC enters into neighboring, uninfected cells, in which it also localizes to the nuclear membrane [18]. SinC of *C*. *psittaci* Cal-10 and the closely related SinC orthologues of *C*. *caviae* GPIC (56% identity to *C*. *psittaci* SinC) and *C*. *abortus* S26/3 (77% identity to *C*. *psittaci* SinC) also localized to the nuclear envelope when expressed as GFP-fusion proteins in uninfected cells [18]. In contrast, a GFP-fusion protein of the more distant SinC orthologue of the human-pathogen *C*. *trachomatis* D/UW-3/CX (CT694; 11% identity to *C*. *psittaci* SinC) did not localize to the nuclear envelope [18], consistent with former studies that proposed that CT694 localizes to the plasma membrane of *C*. *trachomatis*-infected cells [19, 20].

Our ability to characterize *Chlamydia* virulence factors and to study the mechanisms underlying the cross-species transmission and pathogenesis of zoonotic *Chlamydia* species has historically been limited by the genetic intractability of these bacteria. However, in spite of technical difficulties arising from the obligate intracellular and developmental lifestyles of *Chlamydia* spp., in the past decade various genetic techniques have been developed for the human pathogen *C*. *trachomatis* [21]. A major milestone was the implementation of an experimental strategy for transformation of *C*. *trachomatis* with plasmids that mediate heterologous protein expression [22, 23]. More recently, strategies were developed to mediate targeted genetic modifications, such as gene disruptions and gene replacements [24, 25]. The most widely applied technique in this context is TargeTron, which is based on transient transformation of *Chlamydia* with a plasmid that encodes an altered group-II intron and all necessary components for its insertion into a specific gene of interest [24]. TargeTron has been used successfully to perform insertional mutagenesis in *C*. *trachomatis*, with specificity, reproducibility, long-term maintenance in cell culture, and *in vivo* stability [24, 26]. For instance, TargeTron enabled the generation of a *C*. *trachomatis* strain that is deficient for the T3S effector protein IncA, providing molecular genetic evidence of IncA’s role in mediating the fusion of *C*. *trachomatis* inclusions [24]. To date, this genetic tool has not been used in any of the other phylogenetically distinct *Chlamydia* species.

Here, we demonstrate the applicability of the TargeTron system for site-specific mutagenesis in *C*. *caviae* and we investigate the phenotypic properties of *sinC* and *incA* insertion mutants of *C*. *caviae* strain GPIC in cell culture and in a chicken embryo infection model.

## Material and methods

### Cell culture and infection

Vero (ATCC CCL-81), HeLa (ATCC CCL-2), and UMNSAH/DF-1 (ATCC CRL-12203) cells were routinely maintained at 37°C, 5%CO_2_ in Dulbecco's Modified Eagle's Medium (DMEM; Thermo Fisher Scientific or Mediatech) supplemented with 10% heat-inactivated fetal bovine serum (Thermo Fisher Scientific or Atlanta Biologicals). JH4 (ATCC CCL-158) cells were maintained in Ham's F-12K (Kaighn's) Medium (Thermo Fisher Scientific) supplemented with 10% heat-inactivated fetal bovine serum. The wild-type strain of *C*. *caviae* GPIC (parental strain of both *sinC* and *incA* mutants) originated from the laboratory of Roger Rank (University of Arkansas for Medical Sciences). For generation of bacterial preparations used during transformation and in infection experiments, bacteria were propagated in Vero cells, harvested at about 40–48 hpi by H_2_O-mediated lysis and/or sonication of host cells, and titered, as described previously [27, 28]. Bacteria were stored in SPG (sucrose-phosphate-glutamate) buffer (75 g/l sucrose, 0.5 g/l KH_2_PO_4_, 1.2 g/l Na_2_HPO_4_, 0.72 g/l glutamic acid, pH 7.5) at -80°C. Two distinct infection procedures were used. For infection of cells grown in multi-well plates (which was done in all experiments except for the generation of samples for western blot analysis), plates were centrifuged (1500 x g, 30 min, room temperature) after addition of the bacteria. For infection of cells grown in 100 mm dishes (which was done for the preparation of samples for western blot analysis), dishes were rocked at room temperature for 2 h after addition of the bacteria, with additional hand-rocking at 15 min intervals.

### Generation of vector pDFTT3-CAT

To enable gene disruption with an intron carrying a chloramphenicol resistance marker, vector pDFTT3 [24] was modified in a two-step process to generate vector pDFTT3-CAT (S1 and S2 Figs). First, to remove the *cat* gene from the vector backbone, vector pDFTT3 was PCR-amplified (QuikChange II XL Site-Directed Mutagenesis Kit, Agilent) using primers 5’-GTAGGGCCCTTTAGCTTCCTTAGCTCC-3’ and 5’-CCAGGGCCCTAATTTTTTTAAGG CAGT-3’, digested with ApaI (NEB), and circularized by ligation (T4 DNA ligase, NEB). Second, to replace *bla* with *cat* in the intron, this modified vector was further PCR-amplified with primers 5’-GCACATATGCTGTCAGACCAAGTTTACTC-3’ and 5’-GCCGC ATGCACTCTTCCTTTTTCAATATTATTG-3’, digested with NdeI and SphI (NEB), and ligated to a NdeI/SphI-digested gene block containing *cat* (IDT).

### TargeTron mutagenesis

CCA00550 (*incA*) and CCA00062 (*sinC*) in *C*. *caviae* GPIC were disrupted using the TargeTron approach [24], similar to the recently described disruption of CTL0481 (*cpoS*) in *C*. *trachomatis* [27]. The *incA* and *sinC* gene sequences were scanned for potential target insertion sites using the TargeTron^™^ algorithm (Sigma-Aldrich). Among the four target sites closest to the start codon of each gene, the target site with the lowest E-value (*incA*: 0.238, *sinC*: 0.058) was chosen (S1 Table). The primers IBS-incA (5’-AAAAAAGCTTATAATTATCCTTAGGACTCGTGTTGGTGCGCCCAGATAGGGTG-3’), EBS1d-incA (5’-CAGATTGTACAAATGTGGTGATAACAGATAAGTCGTGTTGTCTAACTTACCTTTCTTTGT-3’), EBS2-incA (5’-TGAACGCAAGTTTCTAATTTCGATTAGTCCTCGATAGAGGAAAGTGTCT-3’), and EBS universal (Sigma-Aldrich) were used to retarget vector pDFTT3-CAT for disruption of *incA*. The primers IBS-sinC (5’- AAAAAAGCTTATAATTATCCTTACTATCCCTAG AGGTGCGCCCAGATAGGGTG-3’), EBS1d-sinC (5’-CAGATTGTACAAATGTGGTGA TAACAGATAAGTCCTAGAGCTTAACTTACCTTTCTTTGT-3’), EBS2-sinC (5’-TGAACG CAAGTTTCTAATTTCGGTTGATAGTCGATAGAGGAAAGTGTCT-3’), and EBS universal (Sigma-Aldrich) were used to retarget vector pDFTT3-CAT for disruption of *sinC*. CaCl_2_-mediated transformation of *Chlamydia* [22] was conducted as recently described [27]. Transformants were selected in presence of 0.5 μg/ml chloramphenicol (Sigma-Aldrich), first added at 12 hours post infection (hpi). Transformants were plaque-purified in presence of 1 μg/ml chloramphenicol. Intron insertion at correct target sites was verified by PCR using primers ccSINC-F/R (5’-GCGGGACCCAGTAGAGTTTC-3’ and 5’-GTCACCCCAGTTCCACTTGT-3’) or ccINCA-F/R (5’-TCCCATAATTGAGGGGGCGA-3’ and 5’-ACTTGAGACGGTGTGCCATC-3’) and by sequencing of the resulting PCR products (Eton Bioscience). The mutations are respectively referred to as *sinC*::GII and *incA*::GII.

### Whole-genome sequencing of bacterial strains

Bacterial genomic DNA was isolated from infected Vero cell cultures. In brief, bacteria were harvested from infected cells at 32 hpi by H_2_O-mediated lysis and sonication. Remaining intact cells and host cell nuclei were removed by centrifugation at low speed (1600 rpm, 10 min). Released bacteria were collected by centrifugation at high speed (17,000 x g, 15 min), resuspended in SPG buffer, and treated for 1 h at 37°C with DNase I (NEB) to remove host DNA. Bacteria were then washed once with PBS, resuspended in a small volume of PBS, followed by heat-inactivation of residual DNase I at 75°C for 20 min. Subsequently, bacterial genomic DNA was isolated using the DNeasy Blood and Tissue kit (Qiagen) and was quantified using the Qubit dsDNA BR Assay kit (Thermo Fisher Scientific). Sequencing library preparation (NEBNext DNA Library Prep Kit) and Illumina PE150 sequencing were conducted by Novogene Europe (Cambridge, UK). Furthermore, a basic bioinformatic analysis, including quality control of reads, mapping of reads (using BWA) to the reference genome of *C*. *caviae* GPIC [RefSeq NC_003361.3 (chromosome) and NC_004720.1 (plasmid)], and detection of SNPs and indels (using SAMTOOLS), was conducted by Novogene. A separate bioinformatic analysis was conducted in-house to determine the number and approximate positions of the TargeTron insertions. In brief, a custom reference genome was created, containing the reference genome of *C*. *caviae* GPIC and the two expected insert sequences. The reads were mapped to the genome (un-mated) using STAR version 2.7.1a. The mapped reads were then converted to BED files using BEDTools version 2.27.1. A custom Java program was used to extract read pairs for which either the forward or the reverse read (but not both) mapped to the expected insert sequences. The filtered BED files were read into R version 3.5.1. The forward and reverse read BED tables were then merged by the sequencing read name column. For each pair of reads, the read that mapped to the *Chlamydia* genome was retained. For this read, the average of the from- and to-positions were calculated as x. A histogram of x was plotted, where the bin sizes were set to 1kb. The sequence data were uploaded to ArrayExpress (E-MTAB-8415).

### Generation of antibodies

Affinity-purified polyclonal *C*. *caviae* GPIC SinC-specific peptide antibodies were generated by BioMatik (Cambridge, ON, Canada). Two rabbits were immunized with an N-terminal SinC peptide (residues 20 to 33) and two others were immunized with a C-terminal SinC peptide (residues 249 to 265). Both N- and C-terminal-specific antibodies were combined at a 1:1 ratio for all SinC detection experiments. Polyclonal guinea pig *C*. *caviae* PmpG5 (CCA00282)-specific antibodies were generated by the laboratory of Roger Rank (University of Arkansas for Medical Sciences) according to the method described by Tan et al [29].

### Immunoblot analysis

For the preparation of protein samples for immunoblot analysis, infected Vero cells were collected at 48 hpi by scraping and centrifugation, resuspended in PBS buffer, and lysed by sonication (Sonifier 250, Branson Ultrasonics). Samples were then boiled for 10 min at 100°C in SDS sample buffer (50 mM Tris HCl, 2% SDS, 10% glycerol, 1% β-mercaptoethanol, 12.5 mM EDTA, 0.02% bromophenol blue). Proteins were resolved by SDS-PAGE (12.5% acrylamide; Bio-Rad) and transferred to Hybond-P polyvinylidene fluoride membranes (GE Healthcare). Membranes were blocked overnight at 4°C in PBS-T (PBS with 0.1% Tween 20) containing 5% milk, followed by incubation with primary antibodies for 1 h at 4°C. The following primary antibodies were used in PBS-T containing 5% BSA: polyclonal rabbit-anti-SinC (1:1000, prepared as described above), monoclonal mouse-anti-IncA (1:10, [30]) and polyclonal guinea pig-anti-PmpG5 (1:2000, prepared as described above). Membranes were washed three times with PBS-T and incubated for 1 h with the following fluorophore-conjugated secondary antibodies (Thermo Fisher Scientific) diluted in PBS-T containing 5% BSA: Alexa Fluor 488 conjugated anti-guinea pig antibodies (1:2000), Alexa Fluor 594 conjugated anti-rabbit antibodies (1:2000), and Alexa Fluor 488 conjugated anti-mouse antibodies (1:1000). After three additional wash steps in PBS-T, signals were visualized using a GE Typhoon 8600 Imager.

### Immunofluorescence imaging

For indirect immunofluorescence microscopy analysis, infected cells were fixed at indicated times for 10 min in 100% methanol (pre-chilled, -20°C) or for 20 min in 4% formaldehyde (room temperature). Formaldehyde-fixed cells were permeabilized with 0.2% triton-X-100 in PBS for 15 min. Cells were then incubated for 20–30 min in blocking solution (PBS containing 2–7.5% BSA) and then probed for 1 h with primary antibodies diluted in blocking solution. The following primary antibodies were used: polyclonal guinea pig-anti-PmpG5 (1:2000, prepared as described above), polyclonal rabbit-anti-SinC (1:1000, prepared as described above), monoclonal mouse-anti-IncA (1:1000, [30]), and polyclonal rabbit-anti-Slc1 (1:400, [31]). Cells were washed three times with wash buffer (PBS or PBS containing 0.1% Triton X-100 and 0.1% BSA) and then incubated for 1 h with secondary antibodies diluted in blocking solution. The following secondary antibodies (Thermo Fisher Scientific) were used: Alexa Fluor 488 conjugated anti-guinea pig (1:2000), Alexa Fluor 488 conjugated anti-rabbit (1:1000), and Alexa Fluor 594 conjugated anti-rabbit (1:2000). DNA was stained with DAPI (200–300 ng/ml; Sigma-Aldrich) or Hoechst 33342 (10 μg/ml; Thermo Fisher Scientific). In some applications, cells were stained with HCS CellMask Deep Red Stain (0.5 μg/ml; Thermo Fisher Scientific). Subsequently, cells were washed three times in wash buffer and embedded using Mowiol mounting medium (24% w/v glycerol, 9.6% Mowiol 4.88, 0.1 M Tris-HCl, pH 8.0) or ProLong Glass Antifade Mountant (Thermo Fisher Scientific). All steps in the staining procedure were carried out at room temperature. Fluorescence images were recorded either on a Zeiss Axio Imager Z.1 fluorescence microscope or on a Zeiss Axio Imager Z.2 fluorescence microscope. Both microscopes were equipped with an ApoTome module and operated via the Zen 2 software (Zeiss).

### Quantification of inclusion morphologies

For the quantitative analysis of inclusion morphologies, Vero cells were infected at different MOIs (10, 1, 0.1), fixed at 36 hpi, and prepared for immunofluorescence analysis as described above. Microscopic images were taken from random fields and inclusions were counted and classified by manual inspection. Inclusion morphology was classified as “fusogenic” when the infected cell contained a single smooth or lobular inclusion or up to 3 separate inclusions. Inclusions were classified as “non-fusogenic” when the infected cell contained more than 3 distinct inclusions. In each of the three replicate experiments, for each group, cells from 9 random microscopic fields (including in total about 100–300 cells) were considered for the evaluation.

### Quantification of infectious progeny

For the quantification of infectious progeny, confluent monolayers of Vero or HeLa cells in 96-well plates (“output collection plates”) were infected with the indicated strains at MOI ~1. Output samples (culture supernatants and cell lysates) were collected/prepared at various time points (12, 24, 30, 36, 42, and 48 hpi). For the collection of supernatants, 160 μl culture supernatant (from 200 μl total volume) were transferred to a fresh 96-well plate, supplemented with 40 μl 5x SPG, and stored at -80°C. For the preparation of cell lysates, cells were incubated for 20 min in 160 μl sterile water to allow cell lysis and lysates were then transferred to a fresh 96-well plate, supplemented with 40 μl 5x SPG, and stored at -80°C. In parallel to the infection of the output collection plates, confluent monolayers of Vero cells in 96-well plates (“input titer plates”) were infected with serial dilutions of the same inoculum. These plates were fixed with formaldehyde (as described above) for microscopy and inclusion counting at 28 hpi. For the enumeration of inclusion-forming units (IFUs) in the output samples, confluent monolayers of Vero cells in 96-well plates (“output titer plates”) were infected with serial dilutions of the collected supernatants and cell lysates and fixed for microscopy at 28 hpi. Fixed cells in input and output titer plates were stained with anti-Slc1 antibody and Hoechst (as described above) to detect bacterial inclusions and nuclei, respectively. Inclusion numbers were determined using an automated high-content fluorescence imaging platform (ArrayScan VTI, Thermo Fisher Scientific) operated via the HCS Studio Cell Analysis Software. From the number of inclusions detected in the input and output titer plates, the number of infectious particles formed during one round of infection could be determined (S3 Fig).

### Cell death analysis

For the quantitative assessment of host cell lysis, HeLa cells were infected with indicated *C*. *caviae* strains (MOI 2.5) and the activity of lactate dehydrogenase (LDH) in culture supernatants (an indicator for lytic cell death) was measured at various time points (24, 30, 36, 40, and 48 hpi). Measurements were done using the *in vitro* cytotoxicity kit (Sigma-Aldrich), according to the manufacturer’s instructions. Absorbance was determined at an Infinite M200 plate reader (Tecan). Activity detected in cell-free medium (blank) was subtracted and values were normalized to the activity detected in a total cell lysate.

### Chicken embryo experiments

Leghorn chicken eggs, supplied by a local farmer (Västerbotten, Sweden), were incubated at 37.5°C, 50% humidity and rotated every third hour. On day 4 of incubation, the eggs were candled to identify fertilized eggs. Fertilized eggs were then injected with 50 μl inoculum into the allantoic cavity. The inoculum consisted of *C*. *caviae* preparations (prepared as described above) or mock lysates of uninfected Vero cells (prepared in the same way), diluted in Hank’s Balanced Salt Solution (Thermo Fisher Scientific). A total of 1x10^5^ IFUs (or equivalent amount of mock preparation) were injected per egg. After injection, the injection holes were sealed with paraffin and tape, and the eggs were returned to the incubator and regularly screened for signs of viability or death (movement of the embryo, visibility and morphology of blood vessels), as previously described [32]. Embryos were euthanized by freezing (-20°C) before reaching embryonic developmental day E14. In total, three independent experiments with 4–7 eggs per group and experiment were conducted. Current Swedish legislation states that experimentation with bird embryos in the first two trimesters of embryonic development does not require an ethical approval.

### Statistical analysis

Statistical analysis was performed using the software GraphPad Prism 8.01, using the statistical tests indicated in the figure legends.

## Results

### Generation of TargeTron group-II intron insertion mutants of *C*. *caviae*

To enable molecular genetic characterization of virulence factors in *C*. *caviae* GPIC, we tested the applicability of the TargeTron system for site-specific insertional mutagenesis in this species. For this purpose, the TargeTron vector pDFTT3, which was constructed by Johnson and Fisher for use in *C*. *trachomatis* and drives expression of the GII intron via the *C*. *trachomatis* CTL0655 promoter [24], was retargeted to enable disruption of the genes of interest, *C*. *caviae sinC* and *incA*. Furthermore, we replaced the *bla* (β-lactamase) gene in the intron with a *cat* (chloramphenicol acetyltransferase) gene to enable selection of insertion mutants using chloramphenicol instead of β-lactam antibiotics (S1 and S2 Figs).

**Fig 1:**
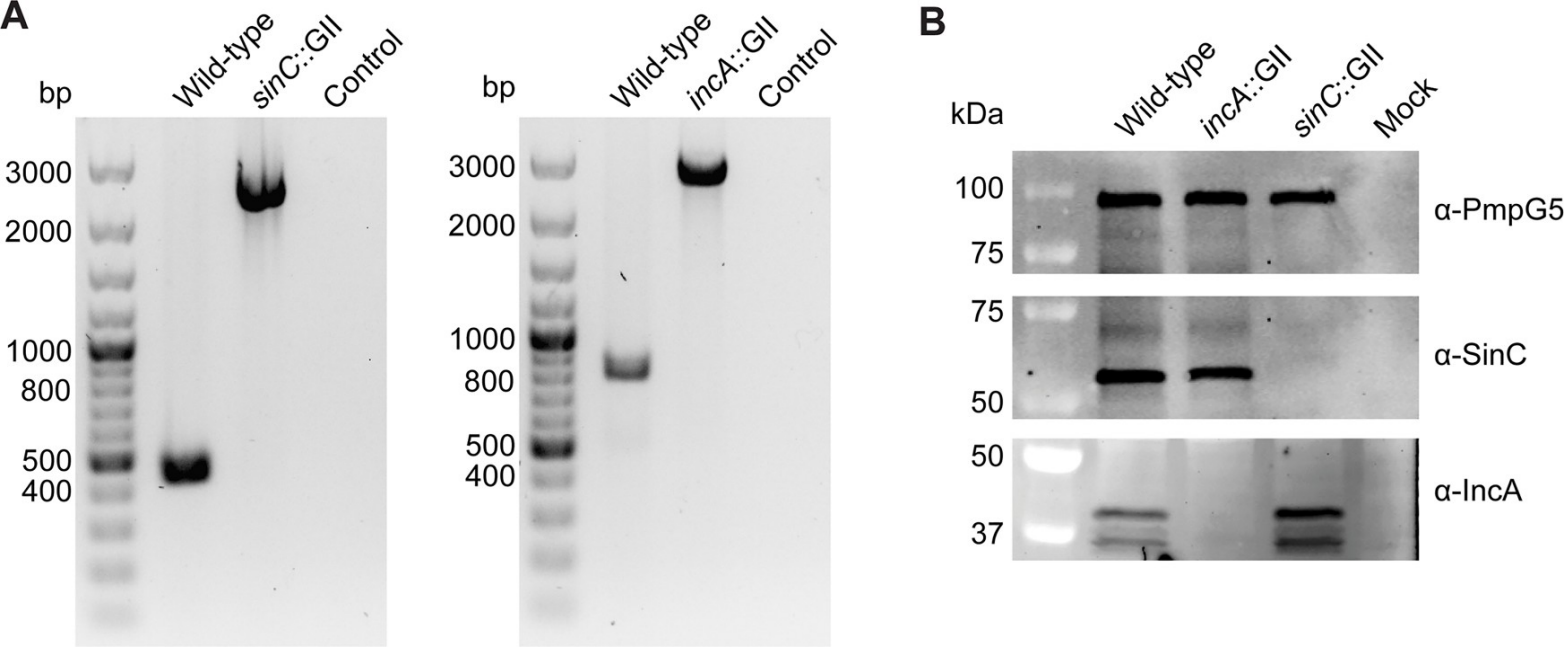
Insertional disruption of *sinC* and *incA* in the *sinC*::GII or *incA*::GII mutants of *C*. *caviae* GPIC. **(A)** PCR-based verification of intron insertion at correct target sites. Primer sets binding to regions flanking either *sinC* (left) or *incA* (right) of *C*. *caviae* GPIC were used to confirm intron insertion at target sites in the respective mutant strains. The primers amplify fragments of 446 bp (*sinC*) and 822 bp (*incA*) in wild-type *C*. *caviae* GPIC in which the genes are intact, and fragments of 2335 bp (*sinC*) and 2711 bp (*incA*) in the mutants in which intron insertion occurred in the respective genes. “Control” refers to the PCR negative control in which only water was used as template. **(B)** Immunoblot analysis confirms absence of SinC and IncA protein in cells infected with *sinC*::GII or *incA*::GII mutants, respectively. Vero cells were infected with wild-type *C*. *caviae* GPIC, the *sinC*::GII or *incA*::GII mutant, or were mock infected. Protein samples were generated at 48 hpi. The representative blots shown were made with the same samples and same sample amounts loaded; IncA staining was conducted on a separate membrane. The calculated molecular masses of detected proteins are approximately 100.4 kDa (PmpG5), 49.4 kDa (SinC), and 38.8 kDa (IncA).

*C*. *caviae* GPIC was transformed with the resulting plasmids according to the CaCl_2_-based transformation protocol developed by Wang and co-workers [22]. After selection, clonal isolates of transformed bacteria were obtained by the plaque method [33]. We were able to confirm stable site-specific insertion of the group-II intron in both the *sinC*::GII and *incA*::GII mutants of *C*. *caviae* (Fig 1A). PCR using primers that bind to regions flanking the coding sequences of the target genes revealed approximately 2 kb larger fragments in the mutants relative to the parent, suggesting that the intron (1889 bp) had inserted in the target genes. Absence of the parental fragments confirmed the purity of the clonal mutant populations isolated after mutagenesis. The insertion of the group-II intron at the anticipated sites was also confirmed by sequence analysis of the PCR products. Intron insertion occurred between nucleotides 17 and 18 in the *incA* gene, and between nucleotides 359 and 360 in the *sinC* gene. Moreover, whole genome sequencing of the two mutant strains and the parental strain confirmed single insertion events of the TargeTron introns into the genomes of the mutants (S4 Fig). A comparison with the reference genome of *C*. *caviae* GPIC also revealed the presence of a synonymous single nucleotide polymorphism (SNP) in the genomes of two of the strains (S2 Table), but no other SNPs or indels were found.

For detection of *C*. *caviae* SinC in immunostaining applications, we generated SinC-specific polyclonal antibodies engineered to recognize highly immunogenic peptides in the N-terminus (residues 20 to 33) or C-terminus (residues 249 to 265) of SinC. The N-terminus specific antibodies were expected to detect both uninterrupted SinC and potential truncates that may be expressed in the *sinC*::GII insertion mutant. Immunoblot analysis of lysates of cells infected with *sinC*::GII revealed no reactivity with these antibodies (Fig 1B). The absence of an immuno-reactive band suggests that *sinC*::GII does either not express truncated SinC at all or that the truncated protein is unstable and degraded. Furthermore, in immunoblots of lysates of cells infected with *incA*::GII, no reactivity with *C*. *caviae* IncA-specific mouse monoclonal antibodies that recognize the C-terminal end of the protein [30] was observed (Fig 1B).

### The *incA*::GII mutant forms non-fusogenic inclusions in cell culture

**Fig 2:**
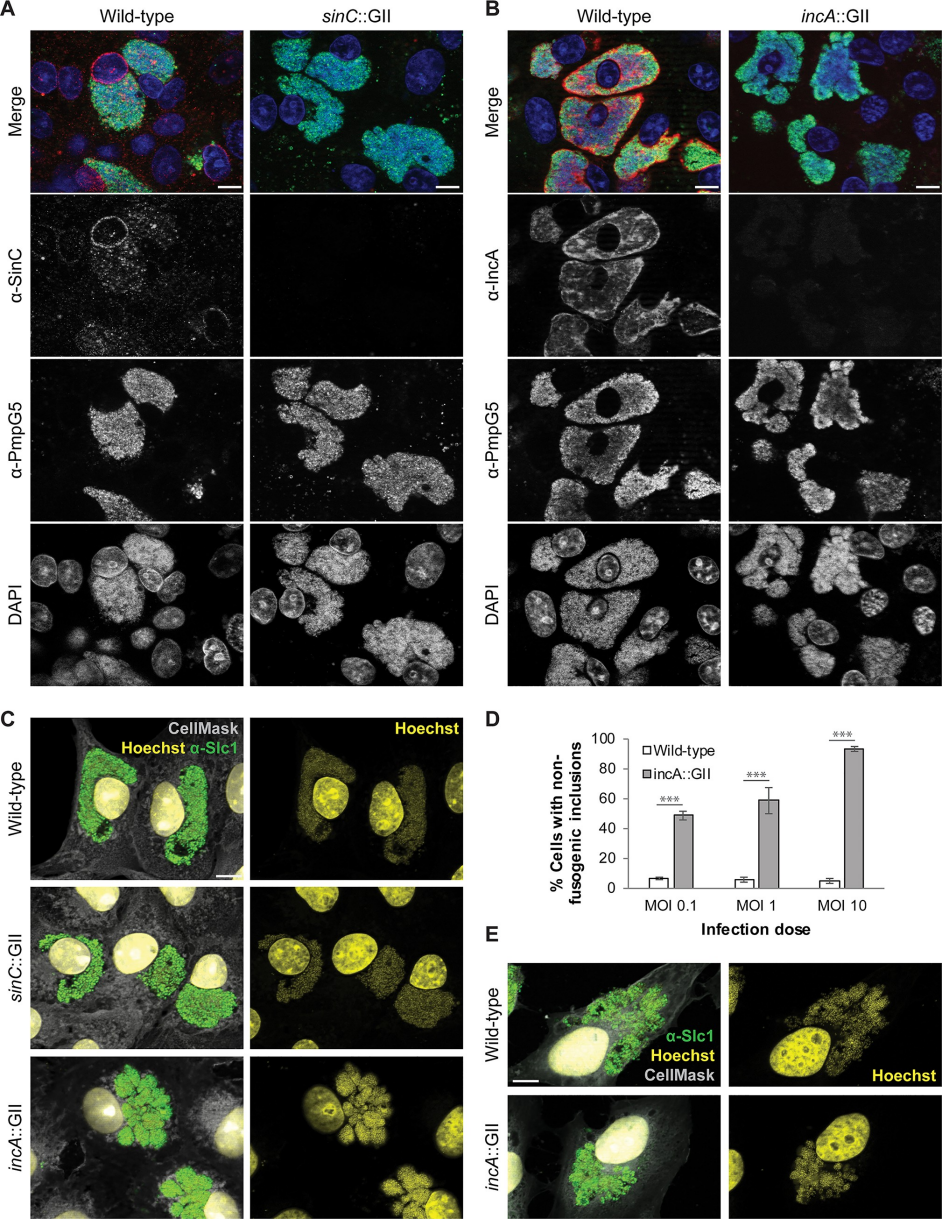
The *C*. *caviae* mutant *incA*::GII forms non-fusogenic inclusions in Vero cells. **(A-B)** Fluorescence microscopic verification of the absence of SinC (A) and IncA (B) in Vero cells infected with respective *C*. *caviae* mutants (MOI 1). Shown are representative micrographs of cells that were fixed and stained at approximately 36 hpi (IncA (red), SinC (red), PmpG5 (green), DAPI (blue); scale bars, 10 μm). **(C)** Visualization of inclusion morphologies in Vero cells infected with wild-type or mutant *C*. *caviae* strains at an elevated multiplicity of infection (MOI 5). Shown are representative micrographs of cells that were fixed and stained at approximately 24 hpi (Slc1 (green), Hoechst (yellow), HCS CellMask (white); scale bars, 10 μm). **(D)** Quantification of distinct inclusion morphologies observed in Vero cells infected with *C*. *caviae incA*::GII. Inclusion morphology in cells infected with indicated strains at indicated MOIs (36 hpi) was manually categorized into “fusogenic’ (≤ 3 inclusions) and “non-fusogenic” (> 3 inclusions). At least 100 cells were analyzed per group and replicate (mean ± SD, n = 3, two-way ANOVA with Sidak’s multiple comparisons test, *** P < 0.001). **(E)** Visualization of inclusion morphologies in HeLa cells infected with wild-type or mutant *C*. *caviae* strains (MOI 5). Shown are representative micrographs of cells that were fixed and stained at approximately 24 hpi (Slc1 (green), Hoechst (yellow), HCS CellMask (white); scale bars, 10 μm).

We next sought to investigate whether the IncA-deficient mutant of *C*. *caviae* had a defect in inclusion fusion, similarly to what has been reported before for IncA-deficient strains of *C*. *trachomatis* [24, 34]. We therefore analyzed infected Vero cells by immunofluorescence microscopy. This also allowed us to confirm the absence of SinC and IncA in cells infected with the respective mutants, because no signals were observed when the cells were stained with the respective antibodies (Fig 2A and 2B). In contrast, in cells infected with wild-type *C*. *caviae*, SinC localized to the nuclear envelope of infected Vero cells, as previously observed for *C*. *psittaci* SinC in infected HeLa cells and for heterologously expressed *C*. *caviae* SinC-GFP in uninfected HEK293T cells [18]. Moreover, in cells infected with wild-type *C*. *caviae*, IncA was observed in the bacteria and at the inclusion membrane, as expected (Fig 2A and 2B).

Wild-type *C*. *caviae* GPIC produced single, large, often lobular inclusions in Vero cells. While inclusions produced by the *sinC*::GII mutant were morphologically indistinguishable from those produced by the parent strain, inclusions produced by the *incA*::GII insertion mutant were clearly distinct with the *incA*::GII mutant producing multiple inclusions in most infected cells (Fig 2C). A quantitative analysis of inclusion morphologies confirmed a prevalence of the large lobular inclusions during infection with wild-type *C*. *caviae*, while the non-fusogenic inclusion phenotype was prevalent in cells infected with the *C*. *caviae* GPIC *incA*::GII mutant (Fig 2D and S3 Table). Interestingly, in HeLa cells, non-fusogenic (or highly lobular) inclusions were observed for all three strains (shown for the wild-type and the *incA*::GII mutant in Fig 2E). This is similar to the multiple inclusion phenotype reported before by Rockey *et al* for *C*. *caviae* GPIC infections in HeLa cells [35], and may suggest that inclusion fusion and/or fission is also modulated by host cell type specific factors. During infection of fibroblast cell lines derived from guinea pigs and chicken, inclusion morphologies were similar as observed in Vero cells, with the *incA*::GII mutant displaying a distinct multiple inclusion phenotype (S5 Fig).

### The *sinC*::GII mutant displays reduced virulence in a chicken embryo infection model

**Fig 3:**
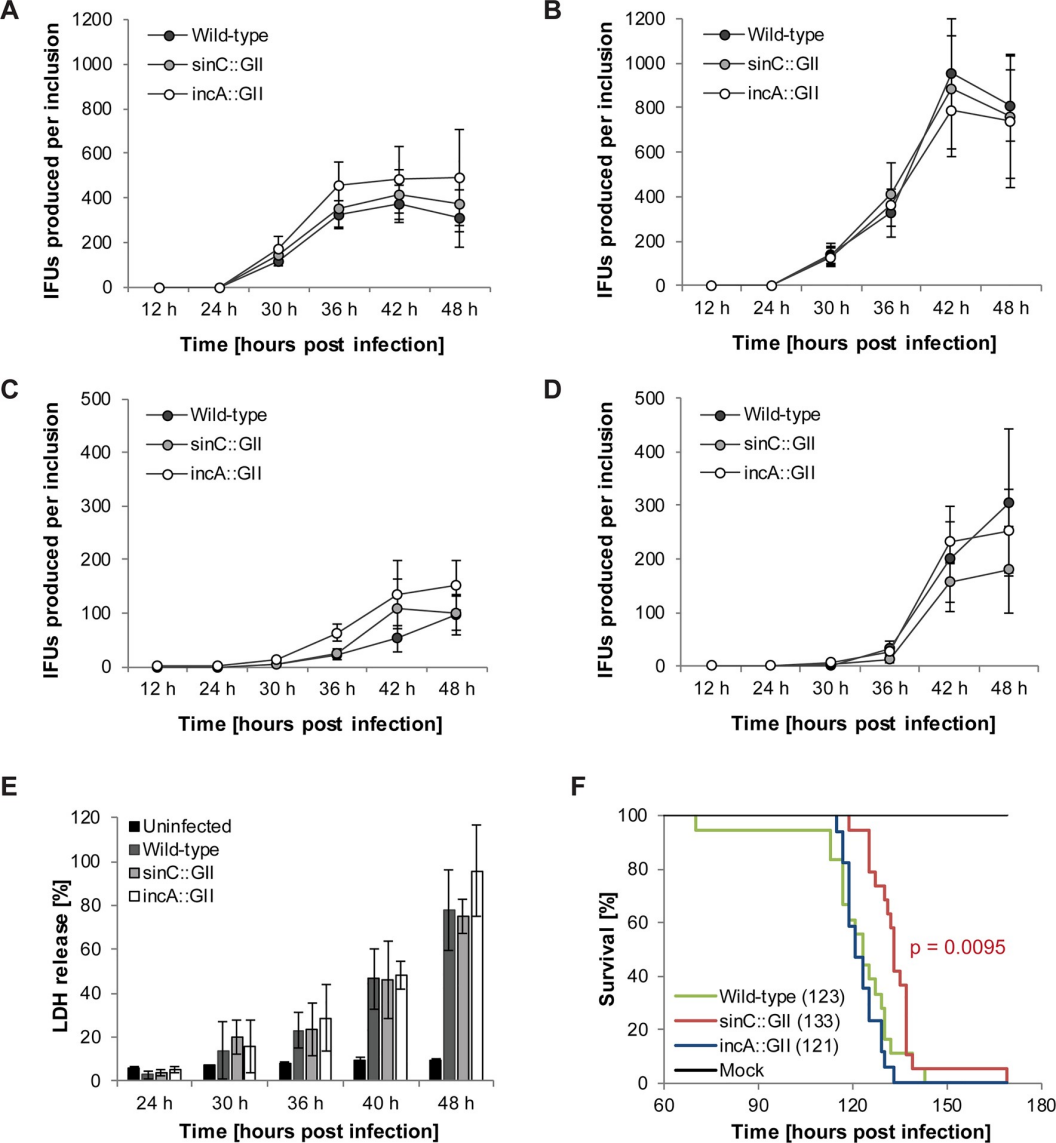
The *C*. *caviae* mutant *sinC*::GII displays reduced virulence in a chicken embryo infection model. **(A-D)** The *C*. *caviae sinC*::GII and *incA*::GII mutants have no growth defect in cell culture. Vero cells (A, C) or HeLa cells (B, D) were infected with the indicated strains (MOI 1) and infectious progeny present in cell lysates (A-B) and culture supernatants (C-D) prepared/collected at the indicated times was quantified in a second round of infection. Results are presented as IFUs produced per inclusion (mean ± SD, n = 3, two-way ANOVA test followed by Tukey’s multiple comparisons; significant differences (p < 0.05) compared to wild-type *C*. *caviae* were not detected at either time). **(E)** The *C*. *caviae sinC*::GII and *incA*::GII mutants have no defect in their ability to induce host cell lysis. HeLa cells were infected with the indicated strains (MOI 2.5). The activity of LDH in culture supernatants (an indicator of lytic host cell death) was measured at indicated times and normalized to the activity detected in a total cell lysate (mean ± SD, n = 3, two-way ANOVA test followed by Tukey’s multiple comparisons; significant differences (p < 0.05) compared to wild-type *C*. *caviae* were not detected at either time). **(F)** Kaplan-Meier survival curve of chicken embryos challenged with *C*. *caviae* displays reduced virulence of the *sinC*::GII mutant. Embryonated chicken eggs were infected with 1x10^5^ IFU/egg *C*. *caviae* [wild-type (n = 18), *sinC*::GII (n = 19), *incA*::GII (n = 17)] or control lysate derived from uninfected cells (n = 19). In the graph legend, median survival is stated in parenthesis for each group. P-values were calculated using Log-rank (Mantel-Cox) test and a Bonferroni-corrected threshold was applied. Consequently, p-values < 0.025 were considered significant.

We next sought to investigate whether deficiency for SinC or IncA affects *C*. *caviae*’s ability to replicate and generate infectious progeny, a hallmark of a completed infection cycle. For this purpose, time course experiments were conducted in which the number of IFUs that could be recovered from infected cell cultures or were released into culture supernatants was quantified at different times post infection. All strains displayed a similar growth curve in cell culture, both in Vero cells and in HeLa cells (Fig 3A–3D and S4–S6 Tables). Moreover, an assessment of the extent of host cell lysis at various times post infection revealed similar profiles for all tested strains (Fig 3E and S7 Table). Cell death was monitored in HeLa cells, because *C*. *caviae* produced higher amounts of infectious progeny in this cell line compared to Vero cells (Fig 3A and 3B). Taken together, the infection experiments in cell culture indicated that all tested strains had a similar capacity not only to produce infectious EBs, but also to complete the infection cycle by inducing the release of the EBs from infected cells.

Because strains that do not have a growth defect in cell culture might still be attenuated *in vivo*, we also compared the virulence of wild-type and mutant *C*. *caviae* strains in a chicken embryo infection model. In this model, fertilized chicken eggs were infected at embryonic development day 4 and the viability of the embryos was monitored in short intervals until day 13. We conducted a total of three independent experiments with 4–7 eggs per group. With the exception of one embryo that died early, eggs infected with wild-type *C*. *caviae* remained viable until about 110 h post infection; afterwards the number of viable embryos started to decline (Fig 3F and S8 Table). At 143 h post infection, all eggs infected with wild-type *C*. *caviae* had died. The median survival was 123 h. In contrast, mock-infected embryos remained viable until day 13, when the experiment was terminated. The *incA*:GII mutant of *C*. *caviae* displayed a slightly (but not statistically significantly) enhanced virulence towards chicken embryos compared to the wild-type strain (median survival 121 h). Interestingly, the *sinC*::GII mutant was considerably attenuated in this infection model (median survival 133 h) (Fig 3F), indicating that SinC is an important effector protein that contributes to *C*. *caviae* virulence *in vivo*.

## Discussion

The successful application of the TargeTron system for the generation of IncA- and SinC-deficient *C*. *caviae* GPIC strains (Fig 1) marks the first time that stable, site-specific mutations have been introduced in a member of the *C*. *caviae-C*. *abortus-C*. *felis*-*C*. *psittaci* lineage. This group of *Chlamydia* is comprised of animal-pathogenic species that have a demonstrated zoonotic potential [7, 8, 12, 36]. Although the GPIC isolate of *C*. *caviae* has never been reported to cause infection in humans in over five decades of laboratory manipulation, recent reports indicate that some *C*. *caviae* strains appear capable of infecting humans and causing respiratory infections that resemble psittacosis [11, 12]. Comparative analyses of the genomic compositions of the GPIC strain and the more recent *C*. *caviae* isolates may in the future aid in identifying genetic differences, such as for instance differences in the repertoire of virulence factors, that could account for the distinct zoonotic potential and pathogenicity of these strains. The ability to generate site-specific mutations in the *C*. *caviae* genome using the TargeTron system will be invaluable in this context for experimentally testing the significance of these genetic differences in *in vivo* infection models and for exploring the molecular mode of action of individual virulence factors in *C*. *caviae*.

IncA was the first *Chlamydia* protein demonstrated to localize to the *Chlamydia* inclusion membrane in infected cells [37]. Today it is known to be a member of a diverse family of *Chlamydia* effector proteins, the so-called inclusion membrane (Inc) proteins, that play a key role in modulating and hijacking host cellular processes to the benefit of the pathogen [38]. Although IncA was first identified in *C*. *caviae* GPIC, most of the current knowledge of IncA function is derived from studies of *C*. *trachomatis* IncA. There is solid evidence that *C*. *trachomatis* IncA mediates fusion between individual *Chlamydia*-containing inclusions within the same cell. First, microinjection of anti-IncA antibodies into *C*. *trachomatis*-infected cells blocked inclusion fusion in cells infected at high MOI [39]. Second, inclusion fusion could first be observed at about 10 hpi and was completed between 18 and 24 hpi, which is consistent with the temporal expression of IncA [39]. Third, occasional natural isolates that display inclusion fusion defects were shown to be deficient for IncA [34]. And fourth, genetic inactivation of *incA* in *C*. *trachomatis* using the TargeTron system resulted in a strain with inclusion fusion defects [24]. The mechanism of IncA-mediated inclusion fusion is not completely understood, yet it has been shown that *C*. *trachomatis* IncA can self-associate to form multimers [39, 40]. It was further proposed that IncA molecules on opposing inclusions could form complexes that resemble SNARE complexes known to mediate membrane fusion events in eukaryotes [40].

Our data demonstrate that *incA* deficiency in *C*. *caviae* has profound effects on inclusion morphology (Fig 2), resulting in an increase in the absolute number of inclusions per cell, similar to what has been observed for *C*. *trachomatis incA* mutants [24] and consistent with a role for *C*. *caviae* IncA in inclusion fusion. IncA of *C*. *caviae* had previously been proposed to be less potent or incapable of mediating inclusion fusion, based on the observation that HeLa cells infected with *C*. *caviae* typically contain multiple and/or highly multi-lobed inclusions [35]. A similar *C*. *caviae* inclusion morphology has been reported for instance in murine L cells [41]. In this study, we found that wild-type *C*. *caviae* formed predominately multiple and/or multi-lobed inclusions in Hela cells, while in Vero cells, as well as in the tested guinea pig and chicken cell lines, most cells infected with wild-type *C*. *caviae* contained only a single large inclusion, similar to those observed in cells infected with *C*. *trachomatis* (Fig 2C and 2E, S5 Fig). Hence, the phenotype caused by IncA deficiency in *C*. *caviae* was easier to detect and quantify in Vero cells than in HeLa cells. Interestingly, in 2000, a study reported that wild-type *C*. *caviae* GPIC formed single large inclusions also in HeLa cells [42], suggesting that inclusion morphology can be influenced by multiple so far unknown factors.

It should also be noted that while infection with *C*. *trachomatis incA* mutants was observed to result in the formation of multiple inclusions per cell only when cells were infected with higher multiplicities of infection [24, 34], in a study reporting a multiple inclusion phenotype for wild-type *C*. *caviae* in HeLa cells, cells infected with *C*. *caviae* developed multiple or multi-lobed inclusions even when infected with a single EB [35]. Moreover, a thorough temporal analysis of *C*. *caviae* inclusion morphology conducted in this study indicated that early bacterial division appeared to be accompanied by inclusion fission leading to the formation of multiple inclusions or lobes [35]. However, from 18 hpi onwards, a time that usually correlates with IncA expression in *Chlamydia* spp. [39], these lobes appeared to expand and to be filled with bacteria [35]. It is thus possible that the occasionally observed distinct inclusion morphologies in cells infected with wild-type *C*. *trachomatis* and *C*. *caviae* may be primarily a consequence of enhanced inclusion fission in *C*. *caviae*-infected cells, as opposed to a lack of fusion. An occurrence of inclusion fission in *C*. *caviae*-infected cells is also supported by our observation that the *incA*::GII mutant produces multiple inclusions per cell in Vero cells even after infection at very low MOI (MOI 0.1) (Fig 2D). The mechanisms of inclusion fission in *Chlamydia*-infected cells and the ways by which host factors may influence inclusion fusion and fission remain to be determined.

The biological relevance of inclusion fusion and fission is currently also unknown. It was shown that among clinical isolates of *C*. *trachomatis*, non-fusogenic strains were associated with milder infections [43]. However, a comparative characterization of matched pairs of IncA-positive and IncA-negative strains isolated from the same patients later indicated that these strains displayed similar growth characteristics in cell culture and in a mouse model of infection [44]. Consistently, an IncA-deficient *C*. *trachomatis* strain generated using the TargeTron procedure displayed a similar ability to produce infectious EBs in cell culture compared to the parental strain [45]. Likewise, we observed that IncA deficiency in *C*. *caviae* did also not negatively affect the ability of the bacteria to produce infectious EBs in cell culture or to induce the release of EBs by host cell lysis (Fig 3A–3E). Furthermore, IncA deficiency in *C*. *caviae* did not significantly affect virulence towards the chicken embryos (Fig 3F). It is possible that in populations of infected individuals, IncA-mediated inclusion fusion may contribute to *Chlamydia* fitness by facilitating genetic exchange between related *Chlamydia* strains infecting the same cell.

The observation that the *sinC*::GII mutant was attenuated in the chicken embryo model highlights the importance of SinC as a virulence factor (Fig 3F). Moreover, our findings suggest that this infection model could be a rapid, inexpensive, and easy set-up for screening of large numbers of *Chlamydia* mutants for *in vivo* virulence defects, similar to what has been done for other pathogens, such as for instance for *Listeria monocytogenes* [32]. The chicken embryo model has also been used to assess the virulence of several other pathogens, such as for instance *Clostridium perfringens* [46], *Staphylococcus aureus* [47], *Escherichia coli* [48], and *Francisella tularensis* [49]. In addition, a recent study assessing *C*. *psittaci* and *C*. *abortus in vivo* virulence has used this model [50]. It should be noted that the chicken embryo model primarily allows to monitor the pathogen’s ability to overcome the vertebrate innate immune defense, because the adaptive immune system of the chicken only starts to develop at day 11 [32]. A more detailed characterization of the *in vivo* virulence defects of the *sinC*::GII mutant thus will need to be conducted in a guinea pig (e.g. [51]) or mouse infection model.

In conclusion, we demonstrate here that GII transposon insertion mutagenesis previously exploited for mutagenesis of the human pathogen *C*. *trachomatis*, is applicable to a phylogenetically distant member of the *Chlamydiaceae*, *C*. *caviae*, a pathogen of the guinea pig. An *incA*::GII mutant displayed a reduced inclusion fusogenicity phenotype in cell culture, confirming the conserved role of IncA across the *Chlamydiaceae*. Moreover, the *in vivo* virulence defect of the *sinC*:GII mutant highlights the importance of this virulence factor for *C*. *caviae*. The broad applicability of the TargeTron method should facilitate the identification and functional analysis of virulence factors from phylogenetically close relatives of *C*. *caviae*, including *C*. *psittaci*, *C*. *abortus* and *C*. *felis*, as well as recent isolates of *C*. *caviae* that can cause life-threatening zoonotic infections in humans.

## Supporting information

S1 Fig Map of vector pDFTT3-CAT. (PDF)

S2 Fig Sequence of vector pDFTT3-CAT. (PDF)

S3 Fig Schematic representation of the procedure used for the quantification of infectious progeny. (PDF)

S4 Fig Whole genome sequencing confirms single insertions of TargeTron introns. (PDF)

S5 Fig The C. caviae mutant incA::GII forms non-fusogenic inclusions in guinea pig and chicken fibroblasts. (PDF)

S1 Table TargeTron target site prediction. (PDF)

S2 Table Detection of SNPs and indels in the genomes of the *C. caviae* strains. (PDF)

S3 Table Quantitative assessment of inclusion morphology. (PDF)

S4 Table Input calculation for the quantification of infectious progeny. (PDF)

S5 Table Output calculation for the quantification of infectious progeny. (PDF)

S6 Table Calculation of IFUs generated per inclusion for the quantification of infectious progeny. (PDF)

S7 Table Quantitative assessment of host cell lysis at late infection stages. (PDF)

S8 Table Monitoring of chicken embryo death and survival. (PDF)

## References

[1] Sachse K, Bavoil PM, Kaltenboeck B, Stephens RS, Kuo CC, Rossello-Mora R, et al Emendation of the family *Chlamydiaceae*: proposal of a single genus, *Chlamydia*, to include all currently recognized species. Syst Appl Microbiol. 2015;38(2):99–103. Epub 2015/01/27. 10.1016/j.syapm.2014.12.004 . 25618261

[2] Ward ME. The chlamydial developmental cycle In: Barron AL, editor. Micobiology of *Chlamydia*. Boca Raton FL: CRC Press; 1988 p. 71–95.

[3] Elwell C, Mirrashidi K, Engel J. *Chlamydia* cell biology and pathogenesis. Nat Rev Microbiol. 2016;14(6):385–400. 10.1038/nrmicro.2016.30 27108705 PMC4886739

[4] Wright HR, Turner A, Taylor HR. Trachoma. Lancet. 2008;371(9628):1945–54. Epub 2008/06/10. S0140-6736(08)60836-3 [pii] 10.1016/S0140-6736(08)60836-3 . 18539226

[5] Newman L, Rowley J, Vander Hoorn S, Wijesooriya NS, Unemo M, Low N, et al Global estimates of the prevalence and incidence of four curable sexually transmitted infections in 2012 based on systematic review and global reporting. PLoS One. 2015;10(12):e0143304 10.1371/journal.pone.0143304 26646541 PMC4672879

[6] Burillo A, Bouza E. Chlamydophila pneumoniae. Infect Dis Clin North Am. 2010;24(1):61–71. Epub 2010/02/23. 10.1016/j.idc.2009.10.002 . 20171546

[7] Longbottom D, Coulter LJ. Animal chlamydioses and zoonotic implications. J Comp Pathol. 2003;128(4):217–44. 10.1053/jcpa.2002.0629 . 12834606

[8] Knittler MR, Sachse K. *Chlamydia psittaci*: update on an underestimated zoonotic agent. Pathogens and disease. 2015;73(1):1–15. Epub 2015/04/09. 10.1093/femspd/ftu007 . 25853998

[9] Beeckman DS, Vanrompay DC. Zoonotic *Chlamydophila psittaci* infections from a clinical perspective. Clin Microbiol Infect. 2009;15(1):11–7. Epub 2009/02/18. 10.1111/j.1469-0691.2008.02669.x . 19220335

[10] Murray ES. Guinea Pig Inclusion Conjunctivitis Virus. I. Isolation and Identification as a Member of the Psittacosis-Lymphogranuloma-Trachoma Group. J Infect Dis. 1964;114:1–12. Epub 1964/02/01. 10.1093/infdis/114.1.1 . 14118043

[11] van Grootveld R, Bilsen MP, Boelsums TL, Heddema ER, Groeneveld GH, Gooskens J, et al *Chlamydia caviae* causing community-acquired pneumonia: an emerging zoonosis. Vector Borne Zoonotic Dis. 2018 Epub 2018/07/10. 10.1089/vbz.2018.2304 . 29985760

[12] Ramakers BP, Heijne M, Lie N, Le TN, van Vliet M, Claessen VPJ, et al Zoonotic *Chlamydia caviae* presenting as community-acquired pneumonia. N Engl J Med. 2017;377(10):992–4. Epub 2017/09/07. 10.1056/NEJMc1702983 . 28877022

[13] Azuma Y, Hirakawa H, Yamashita A, Cai Y, Rahman MA, Suzuki H, et al Genome sequence of the cat pathogen, *Chlamydophila felis*. DNA Res. 2006;13(1):15–23. 10.1093/dnares/dsi027 . 16766509

[14] Voigt A, Schofl G, Saluz HP. The *Chlamydia psittaci* genome: a comparative analysis of intracellular pathogens. PLoS One. 2012;7(4):e35097 Epub 2012/04/17. 10.1371/journal.pone.0035097 22506068 PMC3323650

[15] Thomson NR, Yeats C, Bell K, Holden MT, Bentley SD, Livingstone M, et al The *Chlamydophila abortus* genome sequence reveals an array of variable proteins that contribute to interspecies variation. Genome Res. 2005;15(5):629–40. 10.1101/gr.3684805 . 15837807 PMC1088291

[16] Read TD, Myers GS, Brunham RC, Nelson WC, Paulsen IT, Heidelberg J, et al Genome sequence of *Chlamydophila caviae* (*Chlamydia psittaci* GPIC): examining the role of niche-specific genes in the evolution of the *Chlamydiaceae*. Nucleic Acids Res. 2003;31(8):2134–47. 10.1093/nar/gkg321 . 12682364 PMC153749

[17] Peters J, Wilson DP, Myers G, Timms P, Bavoil PM. Type III secretion à la *Chlamydia*. Trends Microbiol. 2007;15(6):241–51. 10.1016/j.tim.2007.04.005 . 17482820

[18] Mojica SA, Hovis KM, Frieman MB, Tran B, Hsia RC, Ravel J, et al SINC, a type III secreted protein of *Chlamydia psittaci*, targets the inner nuclear membrane of infected cells and uninfected neighbors. Mol Biol Cell. 2015;26(10):1918–34. Epub 2015/03/20. 10.1091/mbc.E14-11-1530 25788290 PMC4436835

[19] Hower S, Wolf K, Fields KA. Evidence that CT694 is a novel Chlamydia trachomatis T3S substrate capable of functioning during invasion or early cycle development. Mol Microbiol. 2009;72(6):1423–37. 10.1111/j.1365-2958.2009.06732.x 19460098 PMC2997736

[20] Bullock HD, Hower S, Fields KA. Domain analyses reveal that *Chlamydia trachomatis* CT694 protein belongs to the membrane-localized family of type III effector proteins. J Biol Chem. 2012;287(33):28078–86. Epub 2012/06/20. 10.1074/jbc.M112.386904 22711538 PMC3431695

[21] Sixt BS, Valdivia RH. Molecular genetic analysis of *Chlamydia* species. Annu Rev Microbiol. 2016;70:179–98. 10.1146/annurev-micro-102215-095539 . 27607551

[22] Wang Y, Kahane S, Cutcliffe LT, Skilton RJ, Lambden PR, Clarke IN. Development of a transformation system for *Chlamydia trachomatis*: restoration of glycogen biosynthesis by acquisition of a plasmid shuttle vector. PLoS Pathog. 2011;7(9):e1002258 Epub 2011/10/04. 10.1371/journal.ppat.1002258 21966270 PMC3178582

[23] Agaisse H, Derre I. A *C*. *trachomatis* cloning vector and the generation of *C*. *trachomatis* strains expressing fluorescent proteins under the control of a *C*. *trachomatis* promoter. PLoS One. 2013;8(2):e57090 10.1371/journal.pone.0057090 23441233 PMC3575495

[24] Johnson CM, Fisher DJ. Site-specific, insertional inactivation of incA in *Chlamydia trachomatis* using a group II intron. PLoS One. 2013;8(12):e83989 10.1371/journal.pone.0083989 24391860 PMC3877132

[25] Mueller KE, Wolf K, Fields KA. Gene deletion by fluorescence-reported allelic exchange mutagenesis in *Chlamydia trachomatis*. mBio. 2016;7(1):e01817–15. 10.1128/mBio.01817-15 26787828 PMC4725004

[26] Lowden NM, Yeruva L, Johnson CM, Bowlin AK, Fisher DJ. Use of aminoglycoside 3' adenyltransferase as a selection marker for *Chlamydia trachomatis* intron-mutagenesis and in vivo intron stability. BMC research notes. 2015;8(1):570 10.1186/s13104-015-1542-9 26471806 PMC4606545

[27] Sixt BS, Bastidas RJ, Finethy R, Baxter RM, Carpenter VK, Kroemer G, et al The *Chlamydia trachomatis* inclusion membrane protein CpoS counteracts STING-mediated cellular surveillance and suicide programs Cell Host Microbe. 2017;21(1):113–21. 10.1016/j.chom.2016.12.002 PMC5233594 28041929

[28] Schachter J, Wyrick PB. Culture and isolation of *Chlamydia trachomatis* Methods in Enzymology. 236: Academic Press; 1994 p. 377–90. 10.1016/0076-6879(94)36028-6 7968623

[29] Tan C, Hsia RC, Shou H, Carrasco JA, Rank RG, Bavoil PM. Variable expression of surface-exposed polymorphic membrane proteins in in vitro-grown *Chlamydia trachomatis*. Cell Microbiol. 2010;12(2):174–87. Epub 2009/10/09. 10.1111/j.1462-5822.2009.01389.x 19811502 PMC3073146

[30] Alzhanov D, Barnes J, Hruby DE, Rockey DD. Chlamydial development is blocked in host cells transfected with *Chlamydophila caviae* incA. BMC microbiology. 2004;4:24 Epub 2004/07/03. 10.1186/1471-2180-4-24 15230981 PMC459217

[31] Chen YS, Bastidas RJ, Saka HA, Carpenter VK, Richards KL, Plano GV, et al The *Chlamydia trachomatis* type III secretion chaperone Slc1 engages multiple early effectors, including TepP, a tyrosine-phosphorylated protein required for the recruitment of CrkI-II to nascent inclusions and innate immune signaling. PLoS Pathog. 2014;10(2):e1003954 10.1371/journal.ppat.1003954 24586162 PMC3930595

[32] Andersson C, Gripenland J, Johansson J. Using the chicken embryo to assess virulence of *Listeria monocytogenes* and to model other microbial infections. Nat Protoc. 2015;10(8):1155–64. Epub 2015/07/03. 10.1038/nprot.2015.073 . 26134955

[33] Banks J, Eddie B, Schachter J, Meyer KF. Plaque formation by *Chlamydia* in L cells. Infect Immun. 1970;1(3):259–62. Epub 1970/03/01. 16557725 10.1128/iai.1.3.259-262.1970 PMC415889

[34] Suchland RJ, Rockey DD, Bannantine JP, Stamm WE. Isolates of *Chlamydia trachomatis* that occupy nonfusogenic inclusions lack IncA, a protein localized to the inclusion membrane. Infect Immun. 2000;68(1):360–7. 10.1128/iai.68.1.360-367.2000 . 10603409 PMC97142

[35] Rockey DD, Fischer ER, Hackstadt T. Temporal analysis of the developing *Chlamydia psittaci* inclusion by use of fluorescence and electron microscopy. Infect Immun. 1996;64(10):4269–78. Epub 1996/10/01. 8926099 10.1128/iai.64.10.4269-4278.1996 PMC174367

[36] Lutz-Wohlgroth L, Becker A, Brugnera E, Huat ZL, Zimmermann D, Grimm F, et al *Chlamydiales* in guinea-pigs and their zoonotic potential. Journal of veterinary medicine. 2006;53(4):185–93. 10.1111/j.1439-0442.2006.00819.x . 16629952

[37] Rockey DD, Heinzen RA, Hackstadt T. Cloning and characterization of a *Chlamydia psittaci* gene coding for a protein localized in the inclusion membrane of infected cells. Mol Microbiol. 1995;15(4):617–26. 10.1111/j.1365-2958.1995.tb02371.x . 7783634

[38] Rockey DD, Scidmore MA, Bannantine JP, Brown WJ. Proteins in the chlamydial inclusion membrane. Microbes Infect. 2002;4(3 SU -):333–40. 11909744 10.1016/s1286-4579(02)01546-0

[39] Hackstadt T, Scidmore-Carlson MA, Shaw EI, Fischer ER. The *Chlamydia trachomatis* IncA protein is required for homotypic vesicle fusion. Cell Microbiol. 1999;1(2):119–30. . 11207546 10.1046/j.1462-5822.1999.00012.x

[40] Delevoye C, Nilges M, Dautry-Varsat A, Subtil A. Conservation of the biochemical properties of IncA from *Chlamydia trachomatis* and *Chlamydia caviae*: oligomerization of IncA mediates interaction between facing membranes. J Biol Chem. 2004;279(45):46896–906. 10.1074/jbc.M407227200 . 15316015

[41] Spears P, Storz J. Biotyping of *Chlamydia psittaci* based on inclusion morphology and response to diethylaminoethyl-dextran and cycloheximide. Infect Immun. 1979;24(1):224–32. Epub 1979/04/01. 457272 10.1128/iai.24.1.224-232.1979 PMC414287

[42] Hsia R, Ohayon H, Gounon P, Dautry-Varsat A, Bavoil PM. Phage infection of the obligate intracellular bacterium, *Chlamydia psittaci* strain guinea pig inclusion conjunctivitis. Microbes Infect. 2000;2(7):761–72. . 10955956 10.1016/s1286-4579(00)90356-3

[43] Geisler WM, Suchland RJ, Rockey DD, Stamm WE. Epidemiology and clinical manifestations of unique *Chlamydia trachomatis* isolates that occupy nonfusogenic inclusions. J Infect Dis. 2001;184(7):879–84. Epub 2001/08/31. 10.1086/323340 . 11528595

[44] Suchland RJ, Jeffrey BM, Xia M, Bhatia A, Chu HG, Rockey DD, et al Identification of concomitant infection with *Chlamydia trachomatis* IncA-negative mutant and wild-type strains by genomic, transcriptional, and biological characterizations. Infect Immun. 2008;76(12):5438–46. Epub 2008/10/15. 10.1128/IAI.00984-08 18852248 PMC2583591

[45] Weber MM, Noriea NF, Bauler LD, Lam JL, Sager J, Wesolowski J, et al A functional core of IncA is required for *Chlamydia trachomatis* inclusion fusion. J Bacteriol. 2016;198(8):1347–55. 10.1128/JB.00933-15 . 26883826 PMC4859576

[46] Alnassan AA, Shehata AA, Kotsch M, Lendner M, Daugschies A, Bangoura B. Embryonated chicken eggs as an alternative model for mixed *Clostridium perfringens* and *Eimeria tenella* infection in chickens. Parasitol Res. 2013;112(6):2299–306. Epub 2013/03/22. 10.1007/s00436-013-3392-5 . 23515571

[47] Polakowska K, Lis MW, Helbin WM, Dubin G, Dubin A, Niedziolka JW, et al The virulence of *Staphylococcus aureus* correlates with strain genotype in a chicken embryo model but not a nematode model. Microbes Infect. 2012;14(14):1352–62. Epub 2012/10/09. 10.1016/j.micinf.2012.09.006 . 23041460

[48] Oh JY, Kang MS, Yoon H, Choi HW, An BK, Shin EG, et al The embryo lethality of *Escherichia coli* isolates and its relationship to the presence of virulence-associated genes. Poult Sci. 2012;91(2):370–5. Epub 2012/01/19. 10.3382/ps.2011-01807 . 22252350

[49] Horzempa J, O'Dee DM, Shanks RM, Nau GJ. *Francisella tularensis* DeltapyrF mutants show that replication in nonmacrophages is sufficient for pathogenesis in vivo. Infect Immun. 2010;78(6):2607–19. Epub 2010/04/14. 10.1128/IAI.00134-10 20385757 PMC2876533

[50] Braukmann M, Sachse K, Jacobsen ID, Westermann M, Menge C, Saluz HP, et al Distinct intensity of host-pathogen interactions in *Chlamydia psittaci*- and *Chlamydia abortus*-infected chicken embryos. Infect Immun. 2012;80(9):2976–88. Epub 2012/06/13. 10.1128/IAI.00437-12 22689815 PMC3418749

[51] Rank RG. In vivo chlamydial infection In: Tan M, Bavoil P, editors. Intracellular Pathogens I: Chlamydiales. Washington, DC: ASM Press; 2012 p. 285–310.

