# Supplementary material for: Insertional mutagenesis in the zoonotic pathogen Chlamydia caviae
Source: PLoS One. 2019 Nov 7;14(11):e0224324. doi: 10.1371/journal.pone.0224324 (PMC6837515; doi:10.1371/journal.pone.0224324)
Supplement: S1 Fig — (PDF) [file pone.0224324.s001.pdf]

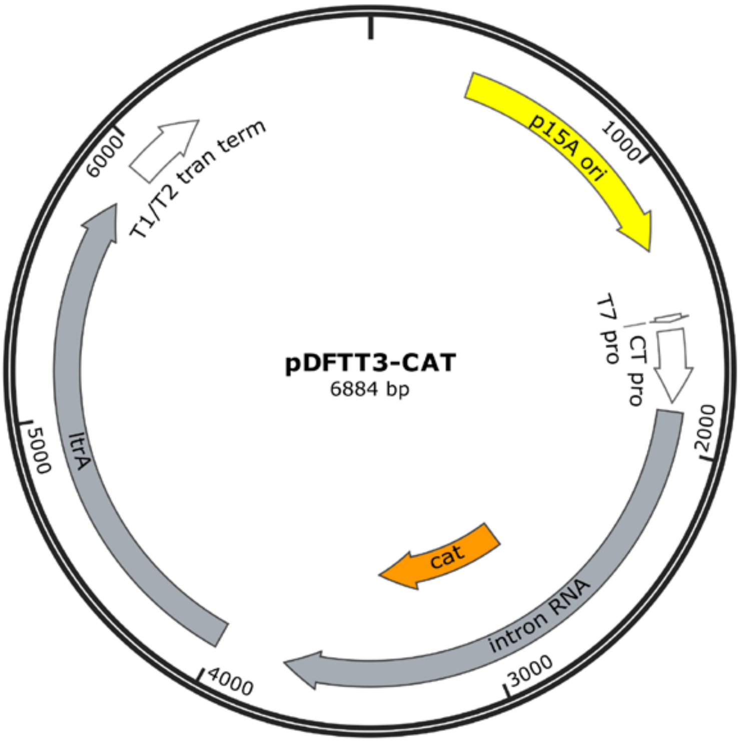

Features

366-1278	p15A ori
1522-1543	T7 promoter
1574-1836	CTL0655 promoter
1868-3756	Intron RNA
2737-3393	cat
4003-5802	ltrA
5938-6228	T1/T2 tran term
1856-1867	IBS
2087-2095	EBS2
2144-2151	EBS1d

S1 Fig: Map of vector pDFTT3-CAT. The vector map was generated using SnapGene software (GSL Biotech). The vector sequence is displayed in S2 Fig.
