# Supplementary material for: Insertional mutagenesis in the zoonotic pathogen Chlamydia caviae
Source: PLoS One. 2019 Nov 7;14(11):e0224324. doi: 10.1371/journal.pone.0224324 (PMC6837515; doi:10.1371/journal.pone.0224324)
Supplement: S2 Fig — (PDF) [file pone.0224324.s002.pdf]

1 tttagcttcc ttagctcctg aaaatctcga taactcaaaa aatacgcccg gtagtgatct
61 tatttcatta tggtgaaagt tggaacctct tacgtgccga tcaacgtctc attttcgcca
121 aaagttggcc cagggcttcc cggtatcaac agggacacca ggatttattt attctgcgaa
181 gtgatcttcc gtcacaggta tttattcggc gcaaagtgcg tcgggtgatg ctgccaactt
241 actgatttag tgtatgatgg tgtttttgag gtgctccagt ggcttctgtt tctatcagct
301 gtccctcctg ttcagctact gacggggtgg tgcgtaacgg caaaagcacc gccggacatc
361 agcgctagcg gagtgtatac tggcttacta tgttggcact gatgagggtg tcagtgaagt
421 gcttcatgtg gcaggagaaa aaaggctgca ccggtgcgtc agcagaatat gtgatacagg
481 atatattccg cttcctcgct cactgactcg ctacgctcgg tcgttcgact gcggcgagcg
541 gaaatggctt acgaacgggg cggagatttc ctggaagatg ccaggaagat acttaacagg
601 gaagtgagag ggccgcggca aagccgtttt tccataggct ccgcccccct gacaagcatc
661 acgaaatctg acgctcaaat cagtggtggc gaaacccgac aggactataa agataccagg
721 cgtttccccc tggcggctcc ctcgtgcgct ctcctgttcc tgcctttcgg tttaccggtg
781 tcattccgct gttatggccg cgtttgtctc attccacgcc tgacactcag ttccgggtag
841 gcagttcgct ccaagctgga ctgtatgcac gaaccccccg ttcagtccga ccgctgcgcc
901 ttatccggta actatcgtct tgagtccaac ccggaaagac atgcaaaagc accactggca
961 gcagccactg gtaattgatt tagaggagtt agtcttgaag tcatgcgccg gttaaggcta
1021 aactgaaagg acaagttttg gtgactgcgc tcctccaagc cagttacctc ggttcaaaga
1081 gttggtagct cagagaacct tcgaaaaacc gccctgcaag gcggtttttt cgttttcaga
1141 gcaagagatt acgcgcagac caaaacgatc tcaagaagat catcttatta atcagataaa
1201 atatttctag ctagatttca gtgcaattta tctcttcaaa tgtagcacct gaagtcagcc
1261 ccatacgata taagttgtaa ttctcatgtt tgacagctta tcatcgataa gctcaaggag
1321 atggcgccca acagtccccc ggccacgggg cctgccacca tacccacgcc gaaacaagcg
1381 ctcatgagcc cgaagtggcg agcccgatct tccccatcgg tgatgtcggc gatataggcg
1441 ccagcaaccg cacctgtggc gccggtgatg ccggccacga tgcgtccggc gtagaggatc
1501 gagatctcga tcccgcgaaa ttaatacgac tcactatagg ggaattgtga gcggataaca
1561 attcccctct agaggtaccg gatcctccaa attattcctt acatgaattt tttgtctgag
1621 cgactttctc ccattgaaaa agattttctt aaacaaaacg tgctttactt cttgcagaaa
1681 aatcggtaga cttgccgttt cgtctaggca gactcgtccg cgtctttttt caaaactccc
1741 tttttaggaa gtttttgaag gcgttcctca gattttcccg agttggagga gactggccgg
1801 cactacaagc tcatatcaag gtaaggaaag atttccaagc ttataattat ccttannnnn
1861 cnnnnnngtg cgcccagata gggtgttaag tcaagtagtt taaggtacta ctctgtaaga
1921 taacacagaa aacagccaac ctaaccgaaa agcgaaagct gatacgggaa cagagcacgg
1981 ttggaaagcg atgagttacc taaagacaat cgggtacgac tgagtcgcaa tgttaatcag
2041 atataaggta taagttgtgt ttactgaacg caagtttcta atttcgnttn nnnnncgata
2101 gaggaaagtg tctgaaacct ctagtacaaa gaaaggtaag ttannnnnnn ngacttatct
2161 gttatcacca catttgtaca atctgtagga gaacctatgg gaacgaaacg aaagcgatgc
2221 cgagaatctg aatttaccaa gacttaacac taactgggga taccctaaac aagaatgcct
2281 aatagaaagg aggaaaaagg ctatagcact agagcttgaa aatcttgcaa gggtacggag
2341 tactcgtagt agtctgagaa gggtaacgcc ctttacatgg caaaggggta cagttattgt
2401 gtactaaaat taaaaattga ttagggagga aaacctcaaa atgaaaccaa caatggcaat
2461 tttagaaaga atcagtaaaa attcacaaga aaatatagac gaagttttta caagacttta
2521 tcgttatctt ttacgtccag atatttatta cgtggcgacg cgtaggttaa tgtcatgata
2581 ataatggttt cttagacgtc aggtggcact tttcggggaa atgtgcgcgg aacccctatt
2641 tgtttatttt tctaaataca ttcaaatatg tatccgctca tgagacaata accctgataa
2701 atgcttcaat aatattgaaa aaggaagagt gcatgcatgg agaaaaaaat cactggatat
2761 accaccgttg atatatccca atggcatcgt aaagaacatt ttgaggcatt tcagtcagtt
2821 gctcaatgta cctataacca gaccgttcag ctggatatta cggccttttt aaagaccgta
2881 aagaaaaata agcacaagtt ttatccggcc tttattcaca ttcttgcccg cctgatgaat
2941 gctcatccgg aattccgtat ggcaatgaaa gacggtgagc tggtgatatg ggatagtgtt
3001 cacccttgtt acaccgtttt ccatgagcaa actgaaacgt tttcatcgct ctggagtgaa
3061 taccacgacg atttccggca gtttctacac atatattcgc aagatgtggc gtgttacggt
3121 gaaaacctgg cctatttccc taaagggttt attgagaata tgtttttcgt ctcagccaat
3181 ccctgggtga gtttcaccag ttttgattta aacgtggcca atatggacaa cttcttcgcc
3241 cccgttttca ccatgggcaa atattatacg caaggcgaca aggtgctgat gccgctggcg
3301 attcaggttc atcatgccgt ctgtgatggc ttccatgtcg gcagaatgct taatgaatta
3361 caacagtact gcgatgagtg gcagggcggg gcgcatatgc tgtcagacca agtttactca
3421 tatatacttt agattgattt aaaacttcat ttttaattta aaaggatcta ggtgaagatc
3481 ctttttgata atctcatgac caaaatccct taacgtgagt tttcgttcca cacgcgttgg
3541 gaaatggcaa tgatagcgaa acaacgtaaa actcttgttg tatgctttca ttgtcatcgt
3601 cacgtgattc ataaacacaa gtgaatgtcg acagtgaatt tttacgaacg aacaataaca
3661 gagccgtata ctccgagagg ggtacgtacg gttcccgaag agggtggtgc aaaccagtca
3721 cagtaatgtg aacaaggcgg tacctcccta cttcaccata tcattttctg cagcccccta
3781 gaaataattt tgtttaactt taagaaggag atatacatat atggctagat cgtccattcc
3841 gacagcatcg ccagtcacta tggcgtgctg ctagcgctat atgcgttgat gcaatttcta
3901 tgcactcgta gtagtctgag aagggtaacg ccctttacat ggcaaagggg tacagttatt
3961 gtgtactaaa attaaaaatt gattagggag gaaaacctca aaatgaaacc aacaatggca
4021 attttagaaa gaatcagtaa aaattcacaa gaaaatatag acgaagtttt tacaagactt

```
4081 tatcgttatc ttttacgtcc agatatttat tacgtggcgt atcaaaattt atattccaat
4141 aaaggagctt ccacaaaagg aatattagat gatacagcgg atggctttag tgaagaaaaa
4201 ataaaaaaga ttattcaatc tttaaaagac ggaacttact atcctcaacc tgtacgaaga
4261 atgtatattg caaaaaagaa ttctaaaaag atgagacctt taggaattcc aactttcaca
4321 gataaattga tccaagaagc tgtgagaata attcttgaat ctatctatga accggtattc
4381 gaagatgtgt ctcacggttt tagacctcaa cgaagctgtc acacagcttt gaaaacaatc
4441 aaaagagagt ttggcggcgc aagatggttt gtggagggag atataaaagg ctgcttcgat
4501 aatatagacc acgttacact cattggactc atcaatctta aaatcaaaga tatgaaaatg
4561 agccaattga tttataaatt tctaaaagca ggttatctgg aaaactggca gtatcacaaa
4621 acttacagcg gaacacctca aggtggaatt ctatctcctc ttttggccaa catctatctt
4681 catgaattgg ataagtttgt tttacaactc aaaatgaagt ttgaccgaga aagtccagaa
4741 agaataacac ctgaatatcg ggagctccac aatgagataa aaagaatttc tcaccgtctc
4801 aagaagttgg agggtgaaga aaaagctaaa gttcttttag aatatcaaga aaaacgtaaa
4861 agattaccca cactcccctg tacctcacag acaaataaag tattgaaata cgtccggtat
4921 gcggacgact tcattatctc tgttaaagga agcaaagagg actgtcaatg gataaaagaa
4981 caattaaaac tttttattca taacaagcta aaaatggaat tgagtgaaga aaaaacactc
5041 atcacacata gcagtcaacc cgctcgtttt ctgggatatg atatacgagt aaggagatct
5101 ggaacgataa aacgatctgg taaagtcaaa aagagaacac tcaatgggag tgtagaactc
5161 cttattcctc ttcaagacaa aattcgtcaa tttatttttg acaagaaaat agctatccaa
5221 aagaaaagata gctcatggtt tccagttcac aggaaatatc ttattcgttc aacagactta
5281 gaaatcatca caatttataa ttctgaactc cgcgggattt gtaattacta cggtctagca
5341 agtaatttta accagctcaa ttattttgct tatcttatgg aatacagctg tctaaaaacg
5401 atagcctcca aacataaggg aacactttca aaaaccattt ccatgtttaa agatggaagt
5461 ggttcgtggg ggatcccgta tgagataaag caaggtaagc agcgccgtta ttttgcaaat
5521 tttagtgaat gtaaatcccc ttatcaattt acggatgaga taagtcaagc tcctgtattg
5581 tatggctatg cccggaatac tcttgaaaac aggttaaaag ctaaatgttg tgaattatgt
5641 gggacgtctg atgaaaatac ttcctatgaa attcaccatg tcaataaggt caaaaatctt
5701 aaaggcaaag aaaaatggga aatggcaatg atagcgaaac aacgtaaaac tcttgttgta
5761 tgctttcatt gtcatcgtca cgtgattcat aaacacaagt gaatgtcgag cacccgttct
5821 cggagcactg tccgaccgct ttggccgccg cccagtcctg ctcgcttcgc tacttggagc
5881 cactatcgac tacgcgatca tggcgaccac acccgtcctg tggatcgcca agctcgccga
5941 tggtagtgtg gggtctcccc atgcgagagt agggaactgc caggcatcaa ataaaacgaa
6001 aggctcagtc gaaagactgg gcctttcgtt ttatctgttg tttgtcggtg aacgctctcc
6061 tgagtaggac aaatccgccg ggagcggatt tgaacgttgc gaagcaacgg cccggagggt
6121 ggcgggcagg acgcccgcca taaactgcca ggcatcaaat taagcagaag gccatcctga
6181 cggatggcct ttttgcgttt ctacaaactc ttcctgtcgt catatctaca agccatcccc
6241 ccacagatac ggtaaactag cctcgttttt gcatcaggaa agcagaacgc catgagcggc
6301 ctcatttctt attctgagtt acaacagtcc gcaccgctgt ccggtagctc cttccggtgg
6361 gcgcggggca tgactatcgt cgccgcactt atgactgtct tctttatcat gcaactcgta
6421 ggacaggtgc cggcagcgcc caacagtccc ccggccacgg ggcctgccac catacccacg
6481 ccgaaacaag cgccctgcac cattatgttc cggatctgca tcgcaggatg ctgctggcta
6541 ccctgtggaa cacctacatc tgtattaacg aagcgctaac cgtttttatc aggctctggg
6601 aggcagaata aatgatcata tcgtcaatta ttacctccac ggggagagcc tgagcaaact
6661 ggcctcaggc atttgagaag cacacggtca cactgcttcc ggtagtcaat aaaccggtaa
6721 accagcaata gacataagcg gctatttaac gaccctgccc tgaaccgacg accgggtcga
6781 atttgctttc gaatttctgc cattcatccg cttattatca cttattcagg cgtagcacca
6841 ggcgtttaag ggcaccaata actgccttaa aaaaattagg gccc
```

S2 Fig: Sequence of vector pDFTT3-CAT. The sequences IBS, EBS2, and EBS1d, which are modified during the process of retargeting the vector for disruption of the gene of interest, are indicated as strings of "n". The exact sequences at these positions in the vector used for disrupting *C. caviae incA* were *ggactcgtgttg* (IBS), *attagtcct* (EBS2), and *gacaacac* (EBS1d). In the vector used for disrupting *C. caviae sinC* these sequences were *ctatccctagag* (IBS), *gttgatagt* (EBS2), and *agctctag* (EBS1d).
