# Supplementary material for: Insertional mutagenesis in the zoonotic pathogen Chlamydia caviae
Source: PLoS One. 2019 Nov 7;14(11):e0224324. doi: 10.1371/journal.pone.0224324 (PMC6837515; doi:10.1371/journal.pone.0224324)
Supplement: S3 Fig — (PDF) [file pone.0224324.s003.pdf]

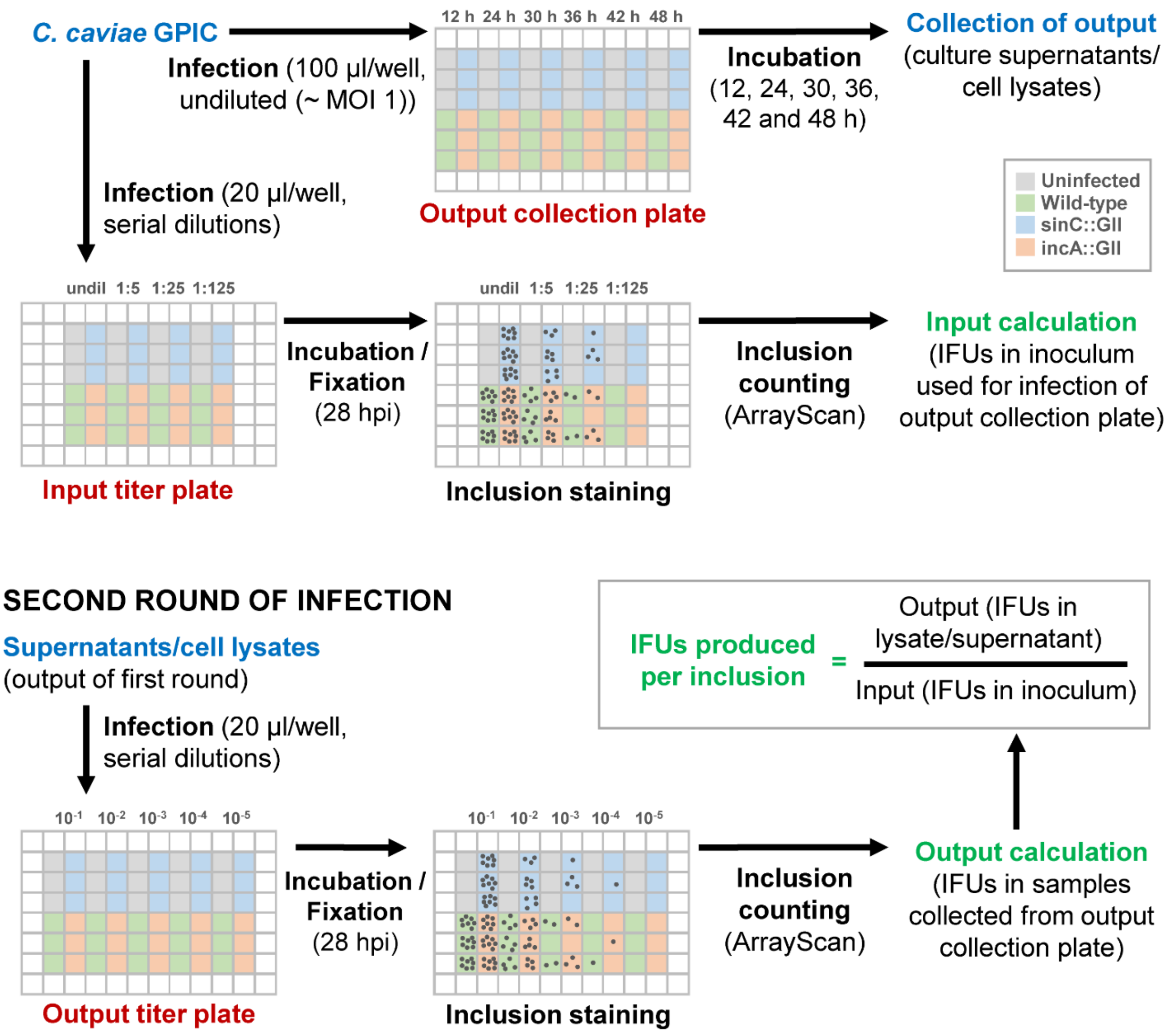

S3 Fig: Schematic representation of the procedure used for the quantification of infectious progeny (see also Figs 3A-D and S4-S6 Tables).
