# Supplementary material for: Insertional mutagenesis in the zoonotic pathogen Chlamydia caviae
Source: PLoS One. 2019 Nov 7;14(11):e0224324. doi: 10.1371/journal.pone.0224324 (PMC6837515; doi:10.1371/journal.pone.0224324)
Supplement: S4 Fig — (PDF) [file pone.0224324.s004.pdf]

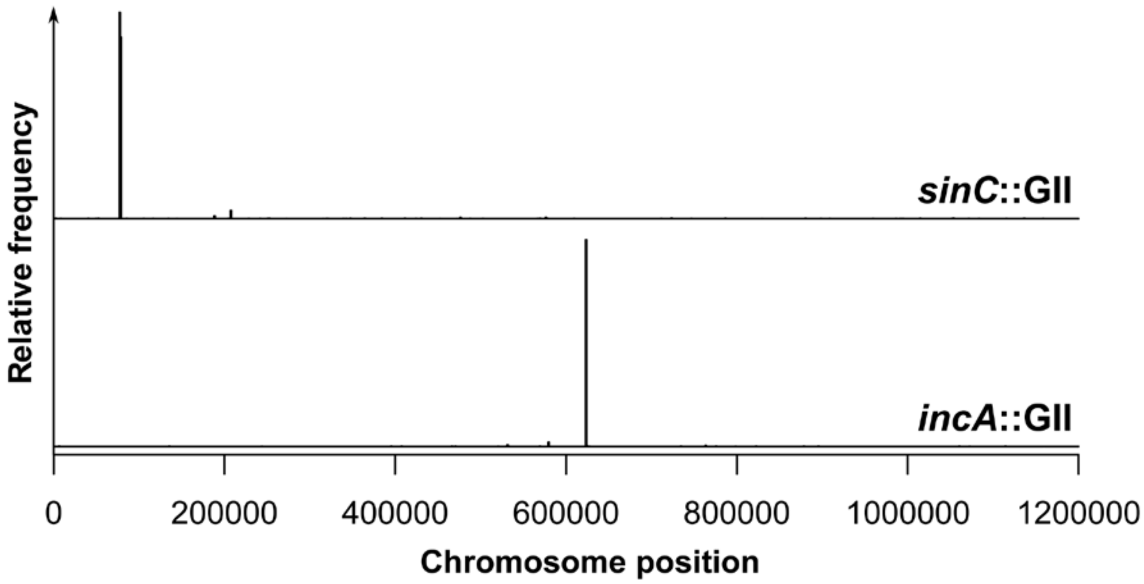

S4 Fig: Whole genome sequencing confirms single insertions of TargeTron introns. After filtering of read pairs in which one paired read maps to the reference genome and the other read maps to the sequence of the TargeTron intron, the average locations of the chromosome-mapping reads of these read pairs along the *Chlamydia* reference genome were displayed as histogram. The two sharp peaks correspond to the expected intron insertion sites. Spurious reads mapped to locations further away from the main peaks can be explained as mapping ambiguities, because additional random insertions would be expected to have the same peak height. Note, that for the wild-type strain reads mapping to the TargeTron intron were not observed.
