# Supplementary material for: Insertional mutagenesis in the zoonotic pathogen Chlamydia caviae
Source: PLoS One. 2019 Nov 7;14(11):e0224324. doi: 10.1371/journal.pone.0224324 (PMC6837515; doi:10.1371/journal.pone.0224324)
Supplement: S5 Fig — (PDF) [file pone.0224324.s005.pdf]

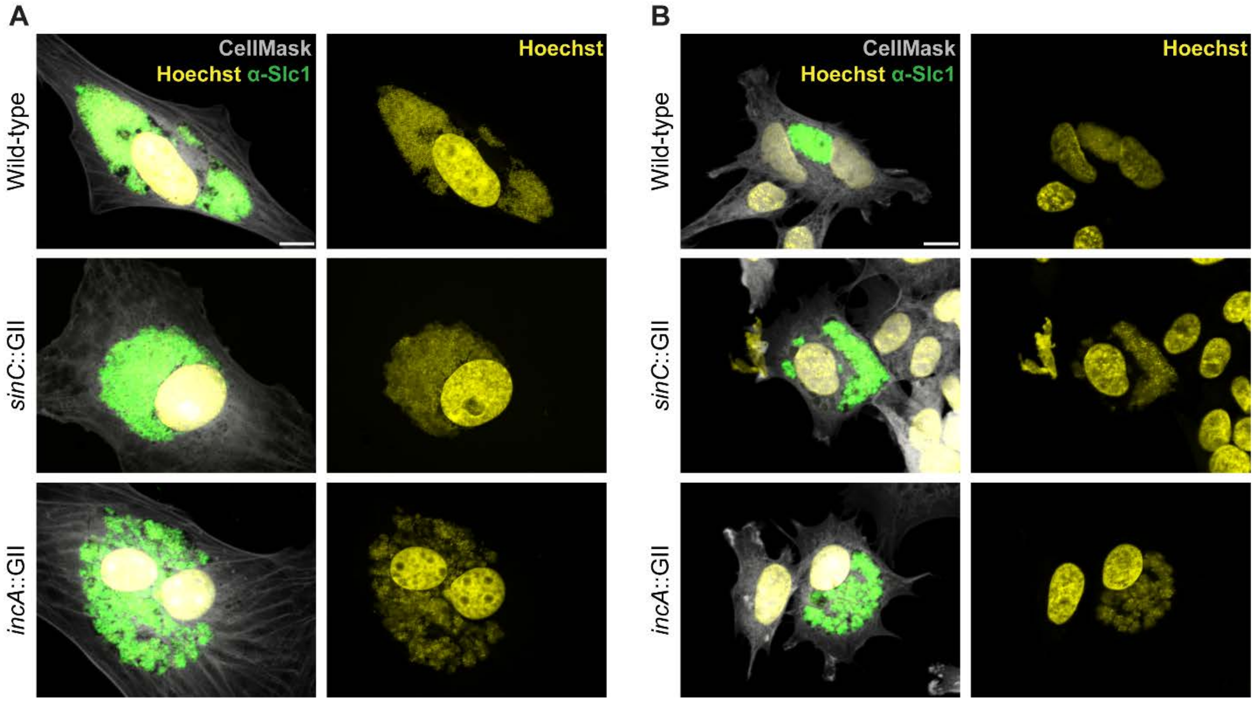

S5 Fig: The *C. caviae* mutant *incA*::GII forms non-fusogenic inclusions in guinea pig and chicken fibroblasts. (A-B) Visualization of inclusion morphologies in JH4 guinea pig cells (A) and UMNSAH/DF-1 chicken cells (B) infected with wild-type or mutant *C. caviae* strains (MOI 5). Shown are representative micrographs of cells that were fixed and stained at 24 hpi (Slc1 (green), Hoechst (yellow), HCS CellMask (white); scale bars, 10 µm).
