# Supplementary material for: Insertional mutagenesis in the zoonotic pathogen Chlamydia caviae
Source: PLoS One. 2019 Nov 7;14(11):e0224324. doi: 10.1371/journal.pone.0224324 (PMC6837515; doi:10.1371/journal.pone.0224324)
Supplement: S1 Table — (PDF) [file pone.0224324.s006.pdf]

S1 Table: TargeTron target site prediction. Displayed are all potential target sites in the *C. caviae* GPIC *sinC* and *incA* genes as predicted by the TargeTron™ algorithm from Sigma-Aldrich. The integration sites marked in bold are the sites used in this study to generate the s*inC*::GII and *incA*::GII mutant strains.

Target site	Sequence (-30 to -1)	Sequence (+1 to +15)	Score	E-value
**Target sites for *sinC***				
143|144a	CTGATTCTTGATAAGAAACCCTGAGTCTCT	GGTGTATTATTTTGT	8.15	0.097
206|207a	TGAGAACTTGTTTGTGAAGAGCTCTTACCA	AATATTCCGGAGAAG	5.88	0.456
**359|360a**	**CCCTCTGTTGATCTTGCACTATCGCTAGAG**	**CTCGTACTTTCTGTT**	**8.84**	**0.058**
454|455a	GGGTATTCGGACGTTGAATTCCTCTACGAG	GAACTCTTGGAGAGT	5.99	0.429
469|470a	GAGGTGGTGGAGGTGGGGTATTCGGACGTT	GAATTCCTCTACGAG	5.88	0.457
559|560s	CAACAAGTGGAACTGGGGTGACAAAACGTA	AGGCTCCTCAACCTC	5.90	0.451
557|558a	GGATCTCCTGCTGGAGGTTGAGGAGCCTTA	CGTTTTGTCACCCCA	7.67	0.140
596|597a	CTAGAAATAGAGGATCCACTTTCATGACGT	GTTCTTTTAGGATCT	7.56	0.151
729|730s	TTAGAAGCTAATAGCGCATCGAGACAAGAG	CGGTTAGAGGCGTTA	5.74	0.493
725|726a	ATCTGACCAGATAACGCCTCTAACCGCTCT	TGTCTCGATGCGCTA	7.70	0.136
730|731a	CTTTAATCTGACCAGATAACGCCTCTAACC	GCTCTTGTCTCGATG	6.17	0.385
838|839a	CTTCTTTCTGAGCATCCGCACGTGCTTGCC	CTAATCGTGCTGTTA	5.92	0.445
880|881a	CCTTTAATGGACGTTCATTAATTCCAGGAC	GCGCTACTTCTACTT	6.53	0.313
901|902a	ATCGAGCTAGAGAAGACGCTGCCTTTAATG	GACGTTCATTAATTC	7.20	0.198
911|912a	TCCCATATAGATCGAGCTAGAGAAGACGCT	GCCTTTAATGGACGT	11.54	0.005
987|988s	CAGAGACAAGATGGGGATTCCGTCCTTCTT	CAAGTGCTGATACGC	6.08	0.407
1032|1033s	ATGGGATTAGAGGGAGGCTTATTAAGCCCT	GAAGTTGACTACGTG	6.55	0.311
1039|1040a	TAACAAGCTGATTTACATAATCCACGTAGT	CAACTTCAGGGCTTA	5.80	0.477
1059|1060s	CCTGAAGTTGACTACGTGGATTATGTAAAT	CAGCTTGTTAGTGAA	7.99	0.110
1084|1085a	TTGTAGGTTGCCAATCGTAATCTTCCTCAG	AATCTCCATATTCAC	6.98	0.232
1119|1120s	GAAGATTACGATTGGCAACCTACAATGCAA	ACTCTTGCTCAAGAT	5.78	0.484
1250|1251a	GTTTTAACCGAAGAAGGCTCATAGTTTCCT	GCATTTGTAGCCATA	6.07	0.408
1302|1303s	GTTAAAACTGCTAATCGTTGGAATGCCGGT	GCTTTGGATTTAATG	6.36	0.346
**Target sites for *incA***				
**17|18a**	**GACGCTCTCGATATTACAGGACTTGTGTTG**	**TCTGTGGATACTGTC**	**6.95**	**0.238**
101|102a	GCTGAAGAAGTTGGAGCTTCTATTGAAATG	GGTATAATTTTATTG	6.32	0.353
190|191s	AGCCTGAAGGAAGAAGCCCACTACTTCAAA	GGATTTGCTATCTTG	6.66	0.292
225|226s	TGCTATCTTGTTAAAATTATCGCTGCCATC	GCCCTCTTTGTTGTT	5.89	0.452
529|530s	TACATACTAGATTATCGGATTTTGGTGATA	GGCTTGAAGCGAATA	6.05	0.414
836|837a	GACAATGTTGAACAAGCCTCTTCTAACTCT	TGTTTACGCTTTTCT	6.78	0.270
850|851a	TCGCAATTGAATGGGACAATGTTGAACAAG	CCTCTTCTAACTCTT	7.00	0.230
