# Supplementary material for: Insertional mutagenesis in the zoonotic pathogen Chlamydia caviae
Source: PLoS One. 2019 Nov 7;14(11):e0224324. doi: 10.1371/journal.pone.0224324 (PMC6837515; doi:10.1371/journal.pone.0224324)
Supplement: S2 Table — (PDF) [file pone.0224324.s007.pdf]

S2 Table: Detection of SNPs and indels in the genomes of the *C. caviae* strains. The table depicts the results of the bioinformatic analysis conducted by Novogene.

Strain	Total reads	Mapped reads	Mapping rate [%]	Average depth [X]	SNP detection	Indel detection
Wild-type	7,836,660	7,705,004	98.32	855	1169337, T>C (synonymous)	none
incA:GII	8,765,998	8,602,986	98.14	950	1169337, T>C (synonymous)	none
sinC:GII	12,686,450	12,436,990	98.03	1352	none	none
