# Supplementary material for: Insertional mutagenesis in the zoonotic pathogen Chlamydia caviae
Source: PLoS One. 2019 Nov 7;14(11):e0224324. doi: 10.1371/journal.pone.0224324 (PMC6837515; doi:10.1371/journal.pone.0224324)
Supplement: S3 Table — (PDF) [file pone.0224324.s008.pdf]

S3 Table: Quantitative assessment of inclusion morphology. The table depicts the raw data underlying the graph displayed in Fig 2D. Inclusion morphology was classified as "fusogenic" when the infected cell contained a single or up to 3 inclusions, and as "non-fusogenic", when the infected cell contained more than 3 distinct inclusions.

**Strain**	**MOI**	**Exp**	**Total cells**	**Fusogenic** [≤ 3 incl/cell]	**Non-fusogenic** [> 3 incl/cell]	**Non-fusogenic [%]** Individual	Mean	SD
Wild-type	0.1	1	283	266	17	6.0		
Wild-type	0.1	2	208	195	13	6.3	**6.5**	0.642
Wild-type	0.1	3	277	257	20	7.2		
Wild-type	1	1	164	153	11	6.7		
Wild-type	1	2	151	142	9	6.0	**5.5**	1.484
Wild-type	1	3	156	150	6	3.8		
Wild-type	10	1	114	109	5	4.4		
Wild-type	10	2	104	101	3	2.9	**4.6**	1.807
Wild-type	10	3	108	101	7	6.5		
incA:GII	0.1	1	129	66	63	48.8		
incA:GII	0.1	2	136	66	70	51.5	**48.5**	3.120
incA:GII	0.1	3	137	75	62	45.3		
incA:GII	1	1	199	87	112	56.3		
incA:GII	1	2	256	125	131	51.2	**58.6**	8.823
incA:GII	1	3	237	75	162	68.4		
incA:GII	10	1	162	13	149	92.0		
incA:GII	10	2	189	10	179	94.7	**93.2**	1.400
incA:GII	10	3	195	14	181	92.8		
