# Supplementary material for: Insertional mutagenesis in the zoonotic pathogen Chlamydia caviae
Source: PLoS One. 2019 Nov 7;14(11):e0224324. doi: 10.1371/journal.pone.0224324 (PMC6837515; doi:10.1371/journal.pone.0224324)
Supplement: S4 Table — (PDF) [file pone.0224324.s009.pdf]

S4 Table: Input calculation for the quantification of infectious progeny. The table depicts a part of the data underlying the graphs displayed in Fig 3A-D (see also S5 and S6 Tables). Confluent monolayers of Vero cells in 96-well plates were infected with serial dilutions of the inoculum that was used to infect the output collection plate (undiluted, 1:5, 1:25, 1:125; 20 µl per well). Cells were fixed at 28 hpi and inclusions were stained and detected using the ArrayScan automated imaging platform. Inclusion counts were used to calculate the number of IFUs present in the inoculum that had been used to infect the output collection plate.

			**Inclusions/field** [a] (corrected for dilution and for false positives)			**Inclusions/well** [b] (inclusions/field x fields/well)			**IFUs in 100 µl inoculum** [c] (inclusions/well x 5)			
**Cells**	**Exp**	**Strain**	**well 1**	**well 2**	**well 3**	**well 1**	**well 2**	**well 3**	**well 1**	**well 2**	**well 3**	**mean**
HeLa	1	Wild-type	62.7	54.4	53.6	4871.4	4231.7	4163.3	$2.4x10^{4}$	2.1 $x10^{4}$	$2.1x10^{4}$	**$2.2x10^{4}$**
HeLa	1	*incA*::GII	72.8	70.2	72.1	5657.3	5458.3	5605.2	$2.8x10^{4}$	2.7 $x10^{4}$	$2.8x10^{4}$	**$2.8x10^{4}$**
HeLa	1	*sinC*::GII	64.1	70.2	66.3	4983.3	5458.3	5155.9	$2.5x10^{4}$	2.7 $x10^{4}$	$2.6x10^{4}$	**$2.6x10^{4}$**
HeLa	2	Wild-type	47.9	42.6	38.3	3722.5	3308.2	2979.4	$1.9x10^{4}$	1.7 $x10^{4}$	$1.5x10^{4}$	**$1.7x10^{4}$**
HeLa	2	*incA*::GII	53.7	52.9	54.1	4171.8	4111.2	4206.0	$2.1x10^{4}$	2.1 $x10^{4}$	$2.1x10^{4}$	**$2.1x10^{4}$**
HeLa	2	*sinC*::GII	54.6	55.2	46.9	4241.0	4292.3	3644.8	$2.1x10^{4}$	2.1 $x10^{4}$	$1.8x10^{4}$	**$2.0x10^{4}$**
HeLa	3	Wild-type	44.7	34.6	38.4	3472.0	2686.1	2988.5	$1.7x10^{4}$	1.3 $x10^{4}$	$1.5x10^{4}$	**$1.5x10^{4}$**
HeLa	3	*incA*::GII	65.7	67.6	61.6	5104.3	5251.2	4784.9	$2.6x10^{4}$	2.6 $x10^{4}$	$2.4x10^{4}$	**$2.5x10^{4}$**
HeLa	3	*sinC*::GII	63.1	58.2	44.3	4905.3	4526.0	3446.3	$2.5x10^{4}$	2.3 $x10^{4}$	$1.7x10^{4}$	**$2.1x10^{4}$**
Vero	1	Wild-type	106.0	101.5	88.6	8239.5	7889.7	6883.9	$4.1x10^{4}$	3.9 $x10^{4}$	$3.4x10^{4}$	**$3.8x10^{4}$**
Vero	1	*incA*::GII	60.9	62.1	87.6	4733.0	4827.9	6806.1	$2.4x10^{4}$	2.4 $x10^{4}$	$3.4x10^{4}$	**$2.7x10^{4}$**
Vero	1	*sinC*::GII	99.0	94.9	89.1	7695.4	7375.9	6926.6	$3.8x10^{4}$	3.7 $x10^{4}$	$3.5x10^{4}$	**$3.7x10^{4}$**
Vero	2	Wild-type	110.6	99.0	99.4	8593.4	7695.6	7729.8	$4.3x10^{4}$	3.8 $x10^{4}$	$3.9x10^{4}$	**$4.0x10^{4}$**
Vero	2	*incA*::GII	78.8	74.6	97.6	6123.1	5795.1	7582.9	$3.1x10^{4}$	2.9 $x10^{4}$	$3.8x10^{4}$	**$3.3x10^{4}$**
Vero	2	*sinC*::GII	116.8	104.4	96.7	9076.9	8118.5	7513.7	$4.5x10^{4}$	4.1 $x10^{4}$	$3.8x10^{4}$	**$4.1x10^{4}$**
Vero	3	Wild-type	110.3	100.3	100.1	8576.3	7799.0	7781.9	$4.3x10^{4}$	3.9 $x10^{4}$	$3.9x10^{4}$	**$4.0x10^{4}$**
Vero	3	*incA*::GII	78.3	74.0	97.2	6088.9	5752.4	7557.3	$3.0x10^{4}$	2.9 $x10^{4}$	$3.8x10^{4}$	**$3.2x10^{4}$**
Vero	3	*sinC*::GII	116.0	104.6	97.3	9017.1	8127.0	7565.8	$4.5x10^{4}$	4.1 $x10^{4}$	$3.8x10^{4}$	**$4.1x10^{4}$**

[a] The number of inclusions/field detected in the optimal dilution was multiplied with the dilution factor. Moreover, the value was corrected for false positives by subtraction of the number of false inclusions detected on average in uninfected wells (< 2 in each experiment).
[b] The number of inclusions/well was calculated by multiplying the number of inclusions/field with the number of fields/well (~77.7).
[c] To calculate the number of IFUs that had been used for infection of the output collection plate, the numbers of inclusions/well had to be multiplied by 5, because a 5 times larger inoculum (100 µl/well) had been used for the infection of the output collection plate.
