# Supplementary material for: Insertional mutagenesis in the zoonotic pathogen Chlamydia caviae
Source: PLoS One. 2019 Nov 7;14(11):e0224324. doi: 10.1371/journal.pone.0224324 (PMC6837515; doi:10.1371/journal.pone.0224324)
Supplement: S5 Table — (PDF) [file pone.0224324.s010.pdf]

S5 Table: Output calculation for the quantification of infectious progeny. The table depicts a part of the data underlying the graphs displayed in Fig 3A-D (see also S4 and S6 Tables). Confluent monolayers of Vero cells in 96-well plates were infected with serial dilutions (10^{-1}, 10^{-2}, 10^{-3}, 10^{-4}, 10^{-5}; 20 µl per well) of the cell lysates/culture supernatants that were prepared/collected at various infection time points (12, 24, 30, 36, 42 and 48 hpi) from the output collection plates. Cells were fixed at 28 hpi and inclusions were stained and detected using the ArrayScan automated imaging platform. Inclusion counts were used to calculate the number of IFUs present in the collected samples.

					**Inclusions/field** [a] (corrected for dilution and for false positives)			**Inclusions/well** [b] (inclusions/field x fields/well)			**IFUs in sample** [c] (lysate = inclusions/well x 10, supernatant = inclusions/well x 10 x.1.5)			
**Cells**	**Sample**	**Exp** [d]	**Strain**	**Time**	**well 1**	**well 2**	**well 3**	**well 1**	**well 2**	**well 3**	**well 1**	**well 2**	**well 3**	**mean**
HeLa	Lysate	1	Wild-type	12 h	4.4	0.0	0.0	344.6	0.0	0.0	3.4×10^{3}	0.0	0.0	**1.1×10^{3}**
HeLa	Lysate	1	*incA*::GII	12 h	0.0	0.0	0.0	0.0	0.0	0.0	0.0	0.0	0.0	**0.0**
HeLa	Lysate	1	*sinC*::GII	12 h	0.0	4.4	0.0	0.0	344.6	0.0	0.0	3.4×10^{3}	0.0	**1.1×10^{3}**
HeLa	Lysate	1	Wild-type	24 h	10.0	6.0	12.5	777.3	466.4	971.6	7.8×10^{3}	4.7×10^{3}	9.7×10^{3}	**7.4×10^{3}**
HeLa	Lysate	1	*incA*::GII	24 h	11.7	11.7	12.0	909.5	909.5	932.8	9.1×10^{3}	9.1×10^{3}	9.3×10^{3}	**9.2×10^{3}**
HeLa	Lysate	1	*sinC*::GII	24 h	8.3	5.7	5.0	645.2	443.1	388.7	6.5×10^{3}	4.4×10^{3}	3.9×10^{3}	**4.9×10^{3}**
HeLa	Lysate	1	Wild-type	30 h	2.7×10^{3}	3.1×10^{3}	2.4×10^{3}	2.1×10^{5}	2.4×10^{5}	1.9×10^{5}	2.1×10^{6}	2.4×10^{6}	1.9×10^{6}	**2.1×10^{6}**
HeLa	Lysate	1	*incA*::GII	30 h	3.1×10^{3}	3.1×10^{3}	4.6×10^{3}	2.4×10^{5}	2.4×10^{5}	3.6×10^{5}	2.4×10^{6}	2.4×10^{6}	3.6×10^{6}	**2.8×10^{6}**
HeLa	Lysate	1	*sinC*::GII	30 h	3.3×10^{3}	3.7×10^{3}	3.4×10^{3}	2.6×10^{5}	2.9×10^{5}	2.6×10^{5}	2.6×10^{6}	2.9×10^{6}	2.6×10^{6}	**2.7×10^{6}**
HeLa	Lysate	1	Wild-type	36 h	5.1×10^{3}	5.1×10^{3}	8.5×10^{3}	4.0×10^{5}	4.0×10^{5}	6.6×10^{5}	4.0×10^{6}	4.0×10^{6}	6.6×10^{6}	**4.8×10^{6}**
HeLa	Lysate	1	*incA*::GII	36 h	9.9×10^{3}	1.1×10^{4}	1.5×10^{4}	7.7×10^{5}	8.8×10^{5}	1.1×10^{6}	7.7×10^{6}	8.8×10^{6}	1.1×10^{7}	**9.3×10^{6}**
HeLa	Lysate	1	*sinC*::GII	36 h	1.0×10^{4}	1.2×10^{4}	1.1×10^{4}	8.1×10^{5}	9.6×10^{5}	8.9×10^{5}	8.1×10^{6}	9.6×10^{6}	8.9×10^{6}	**8.9×10^{6}**
HeLa	Lysate	1	Wild-type	42 h	4.1×10^{4}	3.4×10^{4}	2.3×10^{4}	3.2×10^{6}	2.6×10^{6}	1.8×10^{6}	3.2×10^{7}	2.6×10^{7}	1.8×10^{7}	**2.5×10^{7}**
HeLa	Lysate	1	*incA*::GII	42 h	3.5×10^{4}	3.1×10^{4}	3.9×10^{4}	2.7×10^{6}	2.4×10^{6}	3.0×10^{6}	2.7×10^{7}	2.4×10^{7}	3.0×10^{7}	**2.7×10^{7}**
HeLa	Lysate	1	*sinC*::GII	42 h	4.6×10^{4}	4.0×10^{4}	3.9×10^{4}	3.6×10^{6}	3.1×10^{6}	3.0×10^{6}	3.6×10^{7}	3.1×10^{7}	3.0×10^{7}	**3.2×10^{7}**
HeLa	Lysate	1	Wild-type	48 h	2.8×10^{4}	2.7×10^{4}	2.1×10^{4}	2.2×10^{6}	2.1×10^{6}	1.6×10^{6}	2.2×10^{7}	2.1×10^{7}	1.6×10^{7}	**2.0×10^{7}**
HeLa	Lysate	1	*incA*::GII	48 h	1.3×10^{4}	1.5×10^{4}	2.0×10^{4}	1.0×10^{6}	1.2×10^{6}	1.6×10^{6}	1.0×10^{7}	1.2×10^{7}	1.6×10^{7}	**1.3×10^{7}**
HeLa	Lysate	1	*sinC*::GII	48 h	1.9×10^{4}	1.8×10^{4}	1.3×10^{4}	1.5×10^{6}	1.4×10^{6}	9.8×10^{5}	1.5×10^{7}	1.4×10^{7}	9.8×10^{6}	**1.3×10^{7}**
HeLa	Lysate	2	Wild-type	12 h	4.3	0.0	0.0	336.8	0.0	0.0	3.4×10^{3}	0.0	0.0	**1.1×10^{3}**
HeLa	Lysate	2	*incA*::GII	12 h	0.0	0.0	0.0	0.0	0.0	0.0	0.0	0.0	0.0	**0.0**
HeLa	Lysate	2	*sinC*::GII	12 h	0.0	9.3	0.0	0.0	725.5	0.0	0.0	7.3×10^{3}	0.0	**2.4×10^{3}**
HeLa	Lysate	2	Wild-type	24 h	8.5	7.7	14.9	660.7	598.5	1158.2	6.6×10^{3}	6.0×10^{3}	1.2×10^{4}	**8.1×10^{3}**
HeLa	Lysate	2	*incA*::GII	24 h	10.6	15.1	25.1	823.9	1173.7	1951.0	8.2×10^{3}	1.2×10^{4}	2.0×10^{4}	**1.3×10^{4}**
HeLa	Lysate	2	*sinC*::GII	24 h	25.2	29.6	26.3	1958.8	2300.8	2044.3	2.0×10^{4}	2.3×10^{4}	2.0×10^{4}	**2.1×10^{4}**
HeLa	Lysate	2	Wild-type	30 h	4.2×10^{3}	4.4×10^{3}	3.8×10^{3}	3.2×10^{5}	3.4×10^{5}	3.0×10^{5}	3.2×10^{6}	3.4×10^{6}	3.0×10^{6}	**3.2×10^{6}**

HeLa	Lysate	2	*incA*::GII	30 h	4.4x10^3	4.1x10^3	5.6x10^3	3.4x10^5	3.2x10^5	4.3x10^5	3.4x10^6	3.2x10^6	4.3x10^6	**3.6x10^6**
HeLa	Lysate	2	*sinC*::GII	30 h	5.4x10^3	4.8x10^3	3.8x10^3	4.2x10^5	3.8x10^5	3.0x10^5	4.2x10^6	3.8x10^6	3.0x10^6	**3.7x10^6**
HeLa	Lysate	2	Wild-type	36 h	1.3x10^4	4.4x10^3	4.2x10^3	9.9x10^5	3.4x10^5	3.3x10^5	9.9x10^6	3.4x10^6	3.3x10^6	**5.5x10^6**
HeLa	Lysate	2	*incA*::GII	36 h	5.3x10^3	1.0x10^4	1.3x10^4	4.1x10^5	8.0x10^5	1.0x10^6	4.1x10^6	8.0x10^6	1.0x10^7	**7.4x10^6**
HeLa	Lysate	2	*sinC*::GII	36 h	1.1x10^4	7.9x10^3	6.5x10^3	8.2x10^5	6.2x10^5	5.1x10^5	8.2x10^6	6.2x10^6	5.1x10^6	**6.5x10^6**
HeLa	Lysate	2	Wild-type	42 h	2.2x10^4	1.6x10^4	1.6x10^4	1.7x10^6	1.2x10^6	1.2x10^6	1.7x10^7	1.2x10^7	1.2x10^7	**1.4x10^7**
HeLa	Lysate	2	*incA*::GII	42 h	1.8x10^4	2.1x10^4	2.3x10^4	1.4x10^6	1.6x10^6	1.8x10^6	1.4x10^7	1.6x10^7	1.8x10^7	**1.6x10^7**
HeLa	Lysate	2	*sinC*::GII	42 h	2.1x10^4	1.9x10^4	1.7x10^4	1.6x10^6	1.5x10^6	1.3x10^6	1.6x10^7	1.5x10^7	1.3x10^7	**1.5x10^7**
HeLa	Lysate	2	Wild-type	48 h	1.5x10^4	1.3x10^4	1.2x10^4	1.2x10^6	1.0x10^6	9.4x10^5	1.2x10^7	1.0x10^7	9.4x10^6	**1.0x10^7**
HeLa	Lysate	2	*incA*::GII	48 h	1.9x10^4	1.9x10^4	2.1x10^4	1.4x10^6	1.5x10^6	1.6x10^6	1.4x10^7	1.5x10^7	1.6x10^7	**1.5x10^7**
HeLa	Lysate	2	*sinC*::GII	48 h	2.4x10^4	1.9x10^4	1.4x10^4	1.9x10^6	1.5x10^6	1.1x10^6	1.9x10^7	1.5x10^7	1.1x10^7	**1.5x10^7**
HeLa	Lysate	3	Wild-type	12 h	0.0	0.0	8.3	0.0	0.0	647.8	0.0	0.0	6.5x10^3	**2.2x10^3**
HeLa	Lysate	3	*incA*::GII	12 h	0.0	0.0	0.0	0.0	0.0	0.0	0.0	0.0	0.0	**0.0**
HeLa	Lysate	3	*sinC*::GII	12 h	0.0	0.0	0.0	0.0	0.0	0.0	0.0	0.0	0.0	**0.0x10^0**
HeLa	Lysate	3	Wild-type	24 h	11.5	5.2	4.0	891.3	401.6	308.3	8.9x10^3	4.0x10^3	3.1x10^3	**5.3x10^3**
HeLa	Lysate	3	*incA*::GII	24 h	1.5	9.8	0.2	114.0	759.2	13.0	1.1x10^3	7.6x10^3	1.3x10^2	**3.0x10^3**
HeLa	Lysate	3	*sinC*::GII	24 h	8.3	4.1	0.0	642.6	316.1	0.0	6.4x10^3	3.2x10^3	0.0x10^0	**3.2x10^3**
HeLa	Lysate	3	Wild-type	30 h	2.7 x10^3	2.9 x10^3	2.4 x10^3	2.1x10^5	2.3x10^5	1.9x10^5	2.1x10^6	2.3x10^6	1.9x10^6	**2.1x10^6**
HeLa	Lysate	3	*incA*::GII	30 h	3.0 x10^3	3.0 x10^3	4.4 x10^3	2.3x10^5	2.3x10^5	3.4x10^5	2.3x10^6	2.3x10^6	3.4x10^6	**2.7x10^6**
HeLa	Lysate	3	*sinC*::GII	30 h	3.3 x10^3	3.8 x10^3	3.3 x10^3	2.5x10^5	3.0x10^5	2.6x10^5	2.5x10^6	3.0x10^6	2.6x10^6	**2.7x10^6**
HeLa	Lysate	3	Wild-type	36 h	7.7 x10^3	7.7 x10^3	1.0 x10^4	6.0x10^5	6.0x10^5	7.9x10^5	6.0x10^6	6.0x10^6	7.9x10^6	**6.6x10^6**
HeLa	Lysate	3	*incA*::GII	36 h	1.2 x10^4	1.3 x10^4	1.5 x10^4	9.4x10^5	1.0x10^6	1.2x10^6	9.4x10^6	1.0x10^7	1.2x10^7	**1.0x10^7**
HeLa	Lysate	3	*sinC*::GII	36 h	1.8 x10^4	1.5 x10^4	1.5 x10^4	1.4x10^6	1.2x10^6	1.2x10^6	1.4x10^7	1.2x10^7	1.2x10^7	**1.2x10^7**
HeLa	Lysate	3	Wild-type	42 h	2.1 x10^4	1.5 x10^4	1.6 x10^4	1.6x10^6	1.2x10^6	1.2x10^6	1.6x10^7	1.2x10^7	1.2x10^7	**1.3x10^7**
HeLa	Lysate	3	*incA*::GII	42 h	1.8 x10^4	2.1 x10^4	2.2 x10^4	1.4x10^6	1.6x10^6	1.7x10^6	1.4x10^7	1.6x10^7	1.7x10^7	**1.6x10^7**
HeLa	Lysate	3	*sinC*::GII	42 h	2.1 x10^4	2.0 x10^4	1.6 x10^4	1.6x10^6	1.5x10^6	1.3x10^6	1.6x10^7	1.5x10^7	1.3x10^7	**1.5x10^7**
HeLa	Lysate	3	Wild-type	48 h	2.4 x10^4	2.2 x10^4	7.4 x10^3	1.9x10^6	1.7x10^6	5.8x10^5	1.9x10^7	1.7x10^7	5.8x10^6	**1.4x10^7**
HeLa	Lysate	3	*incA*::GII	48 h	2.9 x10^4	3.3 x10^4	3.9 x10^4	2.3x10^6	2.6x10^6	3.1x10^6	2.3x10^7	2.6x10^7	3.1x10^7	**2.6x10^7**
HeLa	Lysate	3	*sinC*::GII	48 h	3.8 x10^4	3.3 x10^4	1.6 x10^4	3.0x10^6	2.5x10^6	1.2x10^6	3.0x10^7	2.5x10^7	1.2x10^7	**2.2x10^7**
HeLa	Supernatant	1	Wild-type	12 h	10.2	11.9	8.9	790.3	928.6	695.4	9.9x10^3	1.2x10^4	8.7x10^3	**1.0x10^4**
HeLa	Supernatant	1	*incA*::GII	12 h	12.2	13.5	12.1	945.7	1049.1	937.2	1.2x10^4	1.3x10^4	1.2x10^4	**1.2x10^4**
HeLa	Supernatant	1	*sinC*::GII	12 h	9.3	10.5	7.6	721.1	815.9	591.3	9.0x10^3	1.0x10^4	7.4x10^3	**8.9x10^3**
HeLa	Supernatant	1	Wild-type	24 h	5.9	5.7	5.7	458.1	440.2	440.2	5.7x10^3	5.5x10^3	5.5x10^3	**5.6x10^3**
HeLa	Supernatant	1	*incA*::GII	24 h	7.4	9.7	8.6	578.6	751.1	664.9	7.2x10^3	9.4x10^3	8.3x10^3	**8.3x10^3**
HeLa	Supernatant	1	*sinC*::GII	24 h	6.1	5.2	3.3	475.2	406.0	259.1	5.9x10^3	5.1x10^3	3.2x10^3	**4.8x10^3**
HeLa	Supernatant	1	Wild-type	30 h	35.6	33.4	24.5	2.8x10^3	2.6x10^3	1.9x10^3	3.5x10^4	3.2x10^4	2.4x10^4	**3.0x10^4**

HeLa	Supernatant	1	*incA*::GII	30 h	162.2	215.6	247.8	1.3x10^{4}	1.7x10^{4}	1.9x10^{4}	1.6x10^{5}	2.1x10^{5}	2.4x10^{5}	**2.0x10^{5}**
HeLa	Supernatant	1	*sinC*::GII	30 h	87.8	115.6	44.5	6.8x10^{3}	9.0x10^{3}	3.5x10^{3}	8.5x10^{4}	1.1x10^{5}	4.3x10^{4}	**8.0x10^{4}**
HeLa	Supernatant	1	Wild-type	36 h	552.7	470.5	479.4	4.3x10^{4}	3.7x10^{4}	3.7x10^{4}	5.4x10^{5}	4.6x10^{5}	4.7x10^{5}	**4.9x10^{5}**
HeLa	Supernatant	1	*incA*::GII	36 h	554.0	673.8	644.9	4.3x10^{4}	5.2x10^{4}	5.0x10^{4}	5.4x10^{5}	6.5x10^{5}	6.3x10^{5}	**6.1x10^{5}**
HeLa	Supernatant	1	*sinC*::GII	36 h	447.1	270.5	173.8	3.5x10^{4}	2.1x10^{4}	1.4x10^{4}	4.3x10^{5}	2.6x10^{5}	1.7x10^{5}	**2.9x10^{5}**
HeLa	Supernatant	1	Wild-type	42 h	5766.3	966.3	4400.3	4.5x10^{5}	7.5x10^{4}	3.4x10^{5}	5.6x10^{6}	9.4x10^{5}	4.3x10^{6}	**3.6x10^{6}**
HeLa	Supernatant	1	*incA*::GII	42 h	5189.3	6800.3	7100.3	4.0x10^{5}	5.3x10^{5}	5.5x10^{5}	5.0x10^{6}	6.6x10^{6}	6.9x10^{6}	**6.2x10^{6}**
HeLa	Supernatant	1	*sinC*::GII	42 h	3811.3	4411.3	3155.3	3.0x10^{5}	3.4x10^{5}	2.5x10^{5}	3.7x10^{6}	4.3x10^{6}	3.1x10^{6}	**3.7x10^{6}**
HeLa	Supernatant	1	Wild-type	48 h	5881.0	4592.0	4503.0	4.6x10^{5}	3.6x10^{5}	3.5x10^{5}	5.7x10^{6}	4.5x10^{6}	4.4x10^{6}	**4.9x10^{6}**
HeLa	Supernatant	1	*incA*::GII	48 h	6581.0	5758.0	7514.0	5.1x10^{5}	4.5x10^{5}	5.8x10^{5}	6.4x10^{6}	5.6x10^{6}	7.3x10^{6}	**6.4x10^{6}**
HeLa	Supernatant	1	*sinC*::GII	48 h	4814.0	3614.0	2925.0	3.7x10^{5}	2.8x10^{5}	2.3x10^{5}	4.7x10^{6}	3.5x10^{6}	2.8x10^{6}	**3.7x10^{6}**
HeLa	Supernatant	2	Wild-type	12 h	8.7	15.2	9.0	673.9	1183.1	699.6	8.4x10^{3}	1.5x10^{4}	8.7x10^{3}	**1.1x10^{4}**
HeLa	Supernatant	2	*incA*::GII	12 h	12.1	12.2	13.8	941.3	949.9	1071.1	1.2x10^{4}	1.2x10^{4}	1.3x10^{4}	**1.2x10^{4}**
HeLa	Supernatant	2	*sinC*::GII	12 h	8.8	10.1	9.2	682.5	785.9	716.7	8.5x10^{3}	9.8x10^{3}	9.0x10^{3}	**9.1x10^{3}**
HeLa	Supernatant	2	Wild-type	24 h	4.9	4.4	4.2	381.4	338.6	330.1	4.8x10^{3}	4.2x10^{3}	4.1x10^{3}	**4.4x10^{3}**
HeLa	Supernatant	2	*incA*::GII	24 h	3.7	5.0	4.4	286.6	390.0	338.6	3.6x10^{3}	4.9x10^{3}	4.2x10^{3}	**4.2x10^{3}**
HeLa	Supernatant	2	*sinC*::GII	24 h	3.8	2.7	2.8	295.1	208.8	217.4	3.7x10^{3}	2.6x10^{3}	2.7x10^{3}	**3.0x10^{3}**
HeLa	Supernatant	2	Wild-type	30 h	30.0	40.0	21.3	2.3x10^{3}	3.1x10^{3}	1.7x10^{3}	2.9x10^{4}	3.9x10^{4}	2.1x10^{4}	**3.0x10^{4}**
HeLa	Supernatant	2	*incA*::GII	30 h	116.7	151.1	170.0	9.1x10^{3}	1.2x10^{4}	1.3x10^{4}	1.1x10^{5}	1.5x10^{5}	1.7x10^{5}	**1.4x10^{5}**
HeLa	Supernatant	2	*sinC*::GII	30 h	72.2	71.1	47.8	5.6x10^{3}	5.5x10^{3}	3.7x10^{3}	7.0x10^{4}	6.9x10^{4}	4.6x10^{4}	**6.2x10^{4}**
HeLa	Supernatant	2	Wild-type	36 h	567.8	703.3	240.0	4.4x10^{4}	5.5x10^{4}	1.9x10^{4}	5.5x10^{5}	6.8x10^{5}	2.3x10^{5}	**4.9x10^{5}**
HeLa	Supernatant	2	*incA*::GII	36 h	383.3	495.6	542.2	3.0x10^{4}	3.9x10^{4}	4.2x10^{4}	3.7x10^{5}	4.8x10^{5}	5.3x10^{5}	**4.6x10^{5}**
HeLa	Supernatant	2	*sinC*::GII	36 h	263.3	213.3	90.0	2.0x10^{4}	1.7x10^{4}	7.0x10^{3}	2.6x10^{5}	2.1x10^{5}	8.7x10^{4}	**1.8x10^{5}**
HeLa	Supernatant	2	Wild-type	42 h	3444.0	300.0	2844.0	2.7x10^{5}	2.3x10^{4}	2.2x10^{5}	3.3x10^{6}	2.9x10^{5}	2.8x10^{6}	**2.1x10^{6}**
HeLa	Supernatant	2	*incA*::GII	42 h	3578.0	4467.0	4533.0	2.8x10^{5}	3.5x10^{5}	3.5x10^{5}	3.5x10^{6}	4.3x10^{6}	4.4x10^{6}	**4.1x10^{6}**
HeLa	Supernatant	2	*sinC*::GII	42 h	2744.0	3022.0	2422.0	2.1x10^{5}	2.3x10^{5}	1.9x10^{5}	2.7x10^{6}	2.9x10^{6}	2.4x10^{6}	**2.7x10^{6}**
HeLa	Supernatant	2	Wild-type	48 h	4306.0	4761.0	3017.0	3.3x10^{5}	3.7x10^{5}	2.3x10^{5}	4.2x10^{6}	4.6x10^{6}	2.9x10^{6}	**3.9x10^{6}**
HeLa	Supernatant	2	*incA*::GII	48 h	3906.0	4028.0	4117.0	3.0x10^{5}	3.1x10^{5}	3.2x10^{5}	3.8x10^{6}	3.9x10^{6}	4.0x10^{6}	**3.9x10^{6}**
HeLa	Supernatant	2	*sinC*::GII	48 h	2872.0	2872.0	2217.0	2.2x10^{5}	2.2x10^{5}	1.7x10^{5}	2.8x10^{6}	2.8x10^{6}	2.2x10^{6}	**2.6x10^{6}**
HeLa	Supernatant	3	Wild-type	12 h	10.6	8.1	10.7	820.8	630.4	829.4	1.0x10^{4}	7.9x10^{3}	1.0x10^{4}	**9.5x10^{3}**
HeLa	Supernatant	3	*incA*::GII	12 h	17.3	14.0	16.4	1347.1	1088.2	1277.9	1.7x10^{4}	1.4x10^{4}	1.6x10^{4}	**1.5x10^{4}**
HeLa	Supernatant	3	*sinC*::GII	12 h	12.1	12.7	11.7	941.3	984.9	907.1	1.2x10^{4}	1.2x10^{4}	1.1x10^{4}	**1.2x10^{4}**
HeLa	Supernatant	3	Wild-type	24 h	8.7	10.5	9.3	673.9	812.3	725.2	8.4x10^{3}	1.0x10^{4}	9.1x10^{3}	**9.2x10^{3}**
HeLa	Supernatant	3	*incA*::GII	24 h	10.3	11.0	12.5	803.0	855.0	967.8	1.0x10^{4}	1.1x10^{4}	1.2x10^{4}	**1.1x10^{4}**
HeLa	Supernatant	3	*sinC*::GII	24 h	8.9	10.0	10.3	691.0	777.3	803.0	8.6x10^{3}	9.7x10^{3}	1.0x10^{4}	**9.5x10^{3}**
HeLa	Supernatant	3	Wild-type	30 h	46.7	50.0	48.9	3.6x10^{3}	3.9x10^{3}	3.8x10^{3}	4.5x10^{4}	4.9x10^{4}	4.8x10^{4}	**4.7x10^{4}**

HeLa	Supernatant	3	*incA*::GII	30 h	190.0	242.2	274.4	1.5x10^{4}	1.9x10^{4}	2.1x10^{4}	1.8x10^{5}	2.4x10^{5}	2.7x10^{5}	**2.3x10^{5}**
HeLa	Supernatant	3	*sinC*::GII	30 h	115.6	148.9	74.4	9.0x10^{3}	1.2x10^{4}	5.8x10^{3}	1.1x10^{5}	1.4x10^{5}	7.2x10^{4}	**1.1x10^{5}**
HeLa	Supernatant	3	Wild-type	36 h	765.2	851.8	657.4	5.9x10^{4}	6.6x10^{4}	5.1x10^{4}	7.4x10^{5}	8.3x10^{5}	6.4x10^{5}	**7.4x10^{5}**
HeLa	Supernatant	3	*incA*::GII	36 h	685.2	901.8	961.8	5.3x10^{4}	7.0x10^{4}	7.5x10^{4}	6.7x10^{5}	8.8x10^{5}	9.3x10^{5}	**8.3x10^{5}**
HeLa	Supernatant	3	*sinC*::GII	36 h	555.2	415.2	267.4	4.3x10^{4}	3.2x10^{4}	2.1x10^{4}	5.4x10^{5}	4.0x10^{5}	2.6x10^{5}	**4.0x10^{5}**
HeLa	Supernatant	3	Wild-type	42 h	8066.3	1322.3	5344.3	6.3x10^{5}	1.0x10^{5}	4.2x10^{5}	7.8x10^{6}	1.3x10^{6}	5.2x10^{6}	**4.8x10^{6}**
HeLa	Supernatant	3	*incA*::GII	42 h	5722.3	7922.3	7733.3	4.4x10^{5}	6.2x10^{5}	6.0x10^{5}	5.6x10^{6}	7.7x10^{6}	7.5x10^{6}	**6.9x10^{6}**
HeLa	Supernatant	3	*sinC*::GII	42 h	4255.3	5077.3	3833.3	3.3x10^{5}	3.9x10^{5}	3.0x10^{5}	4.1x10^{6}	4.9x10^{6}	3.7x10^{6}	**4.3x10^{6}**
HeLa	Supernatant	3	Wild-type	48 h	8264.3	7142.3	6375.3	6.4x10^{5}	5.6x10^{5}	5.0x10^{5}	8.0x10^{6}	6.9x10^{6}	6.2x10^{6}	**7.1x10^{6}**
HeLa	Supernatant	3	*incA*::GII	48 h	8497.3	8931.3	8864.3	6.6x10^{5}	6.9x10^{5}	6.9x10^{5}	8.3x10^{6}	8.7x10^{6}	8.6x10^{6}	**8.5x10^{6}**
HeLa	Supernatant	3	*sinC*::GII	48 h	7109.3	5653.3	5242.3	5.5x10^{5}	4.4x10^{5}	4.1x10^{5}	6.9x10^{6}	5.5x10^{6}	5.1x10^{6}	**5.8x10^{6}**
Vero	Lysate	1	Wild-type	12 h	6.7	0.0	16.7	518.2	0.0	1295.5	5.2x10^{3}	0.0	1.3x10^{4}	**6.0x10^{3}**
Vero	Lysate	1	*incA*::GII	12 h	0.0	0.0	6.7	0.0	0.0	518.2	0.0	0.0	5.2x10^{3}	**1.7x10^{3}**
Vero	Lysate	1	*sinC*::GII	12 h	6.7	6.7	6.7	518.2	518.2	518.2	5.2x10^{3}	5.2x10^{3}	5.2x10^{3}	**5.2x10^{3}**
Vero	Lysate	1	Wild-type	24 h	20.0	10.0	10.0	1554.6	777.3	777.3	1.6x10^{4}	7.8x10^{3}	7.8x10^{4}	**3.4x10^{4}**
Vero	Lysate	1	*incA*::GII	24 h	0.0	10.0	10.0	0.0	777.3	777.3	0.0	7.8x10^{3}	7.8x10^{4}	**2.9x10^{4}**
Vero	Lysate	1	*sinC*::GII	24 h	20.0	0.0	20.0	1554.6	0.0	1554.6	1.6x10^{4}	0.0	1.6x10^{5}	**5.7x10^{4}**
Vero	Lysate	1	Wild-type	30 h	6.4x10^{3}	7.3x10^{3}	5.8x10^{3}	4.9x10^{5}	5.7x10^{5}	4.5x10^{5}	4.9x10^{6}	5.7x10^{6}	4.5x10^{6}	**5.1x10^{6}**
Vero	Lysate	1	*incA*::GII	30 h	7.5x10^{3}	6.4x10^{3}	8.0x10^{3}	5.8x10^{5}	5.0x10^{5}	6.2x10^{5}	5.8x10^{6}	5.0x10^{6}	6.2x10^{6}	**5.7x10^{6}**
Vero	Lysate	1	*sinC*::GII	30 h	8.1x10^{3}	7.5x10^{3}	6.9x10^{3}	6.3x10^{5}	5.9x10^{5}	5.3x10^{5}	6.3x10^{6}	5.9x10^{6}	5.3x10^{6}	**5.8x10^{6}**
Vero	Lysate	1	Wild-type	36 h	2.3x10^{4}	2.0x10^{4}	7.3x10^{3}	1.8x10^{6}	1.5x10^{6}	5.7x10^{5}	1.8x10^{7}	1.5x10^{7}	5.7x10^{6}	**1.3x10^{7}**
Vero	Lysate	1	*incA*::GII	36 h	1.6x10^{4}	1.8x10^{4}	2.0x10^{4}	1.3x10^{6}	1.4x10^{6}	1.6x10^{6}	1.3x10^{7}	1.4x10^{7}	1.6x10^{7}	**1.4x10^{7}**
Vero	Lysate	1	*sinC*::GII	36 h	3.0x10^{4}	2.3x10^{4}	9.3x10^{3}	2.3x10^{6}	1.8x10^{6}	7.3x10^{5}	2.3x10^{7}	1.8x10^{7}	7.3x10^{6}	**1.6x10^{7}**
Vero	Lysate	1	Wild-type	42 h	1.9x10^{4}	1.8x10^{4}	1.4x10^{4}	1.5x10^{6}	1.4x10^{6}	1.1x10^{6}	1.5x10^{7}	1.4x10^{7}	1.1x10^{7}	**1.3x10^{7}**
Vero	Lysate	1	*incA*::GII	42 h	2.1x10^{4}	1.9x10^{4}	1.9x10^{4}	1.6x10^{6}	1.5x10^{6}	1.5x10^{6}	1.6x10^{7}	1.5x10^{7}	1.5x10^{7}	**1.5x10^{7}**
Vero	Lysate	1	*sinC*::GII	42 h	2.3x10^{4}	1.9x10^{4}	1.5x10^{4}	1.8x10^{6}	1.5x10^{6}	1.2x10^{6}	1.8x10^{7}	1.5x10^{7}	1.2x10^{7}	**1.5x10^{7}**
Vero	Lysate	1	Wild-type	48 h	1.5x10^{4}	9.8x10^{3}	1.1x10^{4}	1.2x10^{6}	7.6x10^{5}	8.3x10^{5}	1.2x10^{7}	7.6x10^{6}	8.3x10^{6}	**9.1x10^{6}**
Vero	Lysate	1	*incA*::GII	48 h	1.8x10^{4}	2.0x10^{4}	1.8x10^{4}	1.4x10^{6}	1.5x10^{6}	1.4x10^{6}	1.4x10^{7}	1.5x10^{7}	1.4x10^{7}	**1.4x10^{7}**
Vero	Lysate	1	*sinC*::GII	48 h	1.8x10^{4}	1.6x10^{4}	1.6x10^{4}	1.4x10^{6}	1.2x10^{6}	1.2x10^{6}	1.4x10^{7}	1.2x10^{7}	1.2x10^{7}	**1.3x10^{7}**
Vero	Lysate	2	Wild-type	12 h	0.0	0.0	0.0	0.0	0.0	0.0	0.0	0.0	0.0	**0.0**
Vero	Lysate	2	*incA*::GII	12 h	0.0	0.0	0.0	0.0	0.0	0.0	0.0	0.0	0.0	**0.0**
Vero	Lysate	2	*sinC*::GII	12 h	0.0	0.0	0.0	0.0	0.0	0.0	0.0	0.0	0.0	**0.0**
Vero	Lysate	2	Wild-type	24 h	0.0	0.0	0.0	0.0	0.0	0.0	0.0	0.0	0.0	**0.0**
Vero	Lysate	2	*incA*::GII	24 h	0.0	0.0	0.0	0.0	0.0	0.0	0.0	0.0	0.0	**0.0**
Vero	Lysate	2	*sinC::GII*	24 h	10.0	10.0	0.0	777.3	777.3	0.0	7.8x10^{3}	7.8x10^{3}	0.0	**5.2x10^{3}**
Vero	Lysate	2	Wild-type	30 h	5.5x10^{3}	5.2x10^{3}	4.2x10^{3}	4.3x10^{5}	4.0x10^{5}	3.3x10^{5}	4.3x10^{6}	4.0x10^{6}	3.3x10^{6}	**3.9x10^{6}**

Vero	Lysate	2	*incA*::GII	30 h	$5.0x10^{3}$	$4.2x10^{3}$	$5.3x10^{3}$	$3.9x10^{5}$	$3.2x10^{5}$	$4.2x10^{5}$	$3.9x10^{6}$	$3.2x10^{6}$	$4.2x10^{6}$	**$3.8x10^{6}$**
Vero	Lysate	2	*sinC*::GII	30 h	$6.0x10^{3}$	$5.6x10^{3}$	$4.8x10^{3}$	$4.7x10^{5}$	$4.4x10^{5}$	$3.7x10^{5}$	$4.7x10^{6}$	$4.4x10^{6}$	$3.7x10^{6}$	**$4.3x10^{6}$**
Vero	Lysate	2	Wild-type	36 h	$1.3x10^{4}$	$1.6\ x10^{4}$	$1.0\ x10^{4}$	$1.0\ x10^{6}$	$1.2\ x10^{6}$	$8.1\ x10^{5}$	$1.0\ x10^{7}$	$1.2\ x10^{7}$	$8.1\ x10^{6}$	**$1.0\ x10^{7}$**
Vero	Lysate	2	*incA*::GII	36 h	$1.4x10^{4}$	$1.3\ x10^{4}$	$1.5\ x10^{4}$	$1.1\ x10^{6}$	$1.0\ x10^{6}$	$1.2\ x10^{6}$	$1.1\ x10^{7}$	$1.0\ x10^{7}$	$1.2\ x10^{7}$	**$1.1\ x10^{7}$**
Vero	Lysate	2	*sinC*::GII	36 h	$1.8x10^{4}$	$1.4\ x10^{4}$	$9.4\ x10^{4}$	$1.4\ x10^{6}$	$1.1\ x10^{6}$	$7.3\ x10^{5}$	$1.4\ x10^{7}$	$1.1\ x10^{7}$	$7.3\ x10^{6}$	**$1.1\ x10^{7}$**
Vero	Lysate	2	Wild-type	42 h	$1.8\ x10^{4}$	$2.3\ x10^{4}$	$7.7x10^{3}$	$1.4x10^{6}$	$1.8x10^{6}$	$6.0x10^{5}$	$1.4x10^{7}$	$1.8x10^{7}$	$6.0x10^{6}$	**$1.3x10^{7}$**
Vero	Lysate	2	*incA*::GII	42 h	$1.6\ x10^{4}$	$1.0\ x10^{4}$	$1.3x10^{4}$	$1.3x10^{6}$	$7.8x10^{5}$	$9.9x10^{5}$	$1.3x10^{7}$	$7.8x10^{6}$	$9.9x10^{6}$	**$1.0x10^{7}$**
Vero	Lysate	2	*sinC*::GII	42 h	$2.0\ x10^{4}$	$2.1\ x10^{4}$	$8.1x10^{3}$	$1.6x10^{6}$	$1.6x10^{6}$	$6.3x10^{5}$	$1.6x10^{7}$	$1.6x10^{7}$	$6.3x10^{6}$	**$1.3x10^{7}$**
Vero	Lysate	2	Wild-type	48 h	$1.3\ x10^{4}$	$1.6\ x10^{4}$	$6.6x10^{3}$	$1.0x10^{6}$	$1.2x10^{6}$	$5.1x10^{5}$	$1.0x10^{7}$	$1.2x10^{7}$	$5.1x10^{6}$	**$9.2x10^{6}$**
Vero	Lysate	2	*incA*::GII	48 h	$1.3\ x10^{4}$	$1.1\ x10^{4}$	$8.2x10^{3}$	$1.0x10^{6}$	$8.8x10^{5}$	$6.4x10^{5}$	$1.0x10^{7}$	$8.8x10^{6}$	$6.4x10^{6}$	**$8.6x10^{6}$**
Vero	Lysate	2	*sinC*::GII	48 h	$1.6\ x10^{4}$	$1.3\ x10^{4}$	$1.3x10^{4}$	$1.3x10^{6}$	$1.0x10^{6}$	$9.9x10^{5}$	$1.3x10^{7}$	$1.0x10^{7}$	$9.9x10^{6}$	**$1.1x10^{7}$**
Vero	Lysate	3	Wild-type	12 h	0.0	10.0	0.0	0.0	777.3	0.0	0.0	$7.8x10^{3}$	0.0	**$2.6x10^{3}$**
Vero	Lysate	3	*incA*::GII	12 h	10.0	10.0	10.0	777.3	777.3	777.3	$7.8x10^{3}$	$7.8x10^{3}$	$7.8x10^{3}$	**$7.8x10^{3}$**
Vero	Lysate	3	*sinC*::GII	12 h	10.0	10.0	10.0	777.3	777.3	777.3	$7.8x10^{3}$	$7.8x10^{3}$	$7.8x10^{3}$	**$7.8x10^{3}$**
Vero	Lysate	3	Wild-type	24 h	0.0	0.0	3.3	0.0	0.0	259.1	0.0	0.0	$2.6x10^{3}$	**$8.6x10^{2}$**
Vero	Lysate	3	*incA*::GII	24 h	0.0	0.0	0.0	0.0	0.0	0.0	0.0	0.0	0.0	**0.0**
Vero	Lysate	3	*sinC*::GII	24 h	0.0	0.0	0.0	0.0	0.0	0.0	0.0	0.0	0.0	**0.0**
Vero	Lysate	3	Wild-type	30 h	$7.1x10^{3}$	$7.3x10^{3}$	$5.3x10^{3}$	$5.5x10^{5}$	$5.6x10^{5}$	$4.1x10^{5}$	$5.5x10^{6}$	$5.6x10^{6}$	$4.1x10^{6}$	**$5.1x10^{6}$**
Vero	Lysate	3	*incA*::GII	30 h	$8.1x10^{3}$	$8.3x10^{3}$	$8.9x10^{3}$	$6.3x10^{5}$	$6.5x10^{5}$	$6.9x10^{5}$	$6.3x10^{6}$	$6.5x10^{6}$	$6.9x10^{6}$	**$6.6x10^{6}$**
Vero	Lysate	3	*sinC*::GII	30 h	$9.3x10^{3}$	$1.0x10^{4}$	$6.0x10^{3}$	$7.2x10^{5}$	$8.1x10^{5}$	$4.6x10^{5}$	$7.2x10^{6}$	$8.1x10^{6}$	$4.6x10^{6}$	**$6.7x10^{6}$**
Vero	Lysate	3	Wild-type	36 h	$2.5x10^{4}$	$1.5x10^{4}$	$2.0x10^{4}$	$1.9x10^{6}$	$1.1x10^{6}$	$1.6x10^{6}$	$1.9x10^{7}$	$1.1x10^{7}$	$1.6x10^{7}$	**$1.5x10^{7}$**
Vero	Lysate	3	*incA*::GII	36 h	$2.1x10^{4}$	$2.2x10^{4}$	$2.2x10^{4}$	$1.7x10^{6}$	$1.7x10^{6}$	$1.7x10^{6}$	$1.7x10^{7}$	$1.7x10^{7}$	$1.7x10^{7}$	**$1.7x10^{7}$**
Vero	Lysate	3	*sinC*::GII	36 h	$2.5x10^{4}$	$1.4x10^{4}$	$1.9x10^{4}$	$2.0x10^{6}$	$1.1x10^{6}$	$1.5x10^{6}$	$2.0x10^{7}$	$1.1x10^{7}$	$1.5x10^{7}$	**$1.5x10^{7}$**
Vero	Lysate	3	Wild-type	42 h	$2.4x10^{4}$	$3.0x10^{4}$	$1.9x10^{4}$	$1.8x10^{6}$	$2.3x10^{6}$	$1.5x10^{6}$	$1.8x10^{7}$	$2.3x10^{7}$	$1.5x10^{7}$	**$1.9x10^{7}$**
Vero	Lysate	3	*incA*::GII	42 h	$2.9x10^{4}$	$2.2x10^{4}$	$2.2x10^{4}$	$2.2x10^{6}$	$1.7x10^{6}$	$1.7x10^{6}$	$2.2x10^{7}$	$1.7x10^{7}$	$1.7x10^{7}$	**$1.9x10^{7}$**
Vero	Lysate	3	*sinC*::GII	42 h	$2.7x10^{4}$	$3.2x10^{4}$	$2.5x10^{4}$	$2.1x10^{6}$	$2.5x10^{6}$	$1.9x10^{6}$	$2.1x10^{7}$	$2.5x10^{7}$	$1.9x10^{7}$	**$2.2x10^{7}$**
Vero	Lysate	3	Wild-type	48 h	$2.4x10^{4}$	$2.6x10^{4}$	$2.1x10^{4}$	$1.9x10^{6}$	$2.0x10^{6}$	$1.6x10^{6}$	$1.9x10^{7}$	$2.0x10^{7}$	$1.6x10^{7}$	**$1.8x10^{7}$**
Vero	Lysate	3	*incA*::GII	48 h	$2.4x10^{4}$	$3.0x10^{4}$	$3.2x10^{4}$	$1.9x10^{6}$	$2.3x10^{6}$	$2.5x10^{6}$	$1.9x10^{7}$	$2.3x10^{7}$	$2.5x10^{7}$	**$2.2x10^{7}$**
Vero	Lysate	3	*sinC*::GII	48 h	$2.9x10^{4}$	$2.4x10^{4}$	$2.6x10^{4}$	$2.3x10^{6}$	$1.9x10^{6}$	$2.0x10^{6}$	$2.3x10^{7}$	$1.9x10^{7}$	$2.0x10^{7}$	**$2.1x10^{7}$**
Vero	Supernatant	1a	Wild-type	12 h	8.9	6.2	7.1	690.8	484.0	552.4	$8.6x10^{3}$	$6.1x10^{3}$	$6.9x10^{3}$	**$7.2x10^{3}$**
Vero	Supernatant	1a	*incA*::GII	12 h	9.3	11.3	10.9	725.0	880.4	846.2	$9.1x10^{3}$	$1.1x10^{4}$	$1.1x10^{4}$	**$1.0x10^{4}$**
Vero	Supernatant	1a	*sinC*::GII	12 h	5.5	6.0	6.0	431.1	466.9	466.9	$5.4x10^{3}$	$5.8x10^{3}$	$5.8x10^{3}$	**$5.7x10^{3}$**
Vero	Supernatant	1a	Wild-type	24 h	5.1	3.5	5.3	395.7	274.4	408.1	$4.9x10^{3}$	$3.4x10^{3}$	$5.1x10^{3}$	**$4.5x10^{3}$**
Vero	Supernatant	1a	*incA*::GII	24 h	7.0	4.2	5.5	544.9	325.7	429.9	$6.8x10^{3}$	$4.1x10^{3}$	$5.4x10^{3}$	**$5.4x10^{3}$**
Vero	Supernatant	1a	*sinC*::GII	24 h	7.5	6.8	3.1	585.3	524.7	240.2	$7.3x10^{3}$	$6.6x10^{3}$	$3.0x10^{3}$	**$5.6x10^{3}$**
Vero	Supernatant	1a	Wild-type	30 h	236.7	240.0	238.9	$1.8x10^{4}$	$1.9x10^{4}$	$1.9x10^{4}$	$2.3x10^{5}$	$2.3x10^{5}$	$2.3x10^{5}$	**$2.3x10^{5}$**

Vero	Supernatant	1a	*incA*::GII	30 h	548.9	552.2	253.3	$4.3x10^{4}$	$4.3x10^{4}$	$2.0x10^{4}$	$5.3x10^{5}$	$5.4x10^{5}$	$2.5x10^{5}$	**$4.4x10^{5}$**
Vero	Supernatant	1a	*sinC*::GII	30 h	165.6	117.8	250.0	$1.3x10^{4}$	$9.2x10^{3}$	$1.9x10^{4}$	$1.6x10^{5}$	$1.1x10^{5}$	$2.4x10^{5}$	**$1.7x10^{5}$**
Vero	Supernatant	1a	Wild-type	36 h	841.3	819.3	808.3	$6.5x10^{4}$	$6.4x10^{4}$	$6.3x10^{4}$	$8.2x10^{5}$	$8.0x10^{5}$	$7.9x10^{5}$	**$8.0x10^{5}$**
Vero	Supernatant	1a	*incA*::GII	36 h	3052.3	3263.3	1097.3	$2.4x10^{5}$	$2.5x10^{5}$	$8.5x10^{4}$	$3.0x10^{6}$	$3.2x10^{6}$	$1.1x10^{6}$	**$2.4x10^{6}$**
Vero	Supernatant	1a	*sinC*::GII	36 h	775.3	352.3	1575.3	$6.0x10^{4}$	$2.7x10^{4}$	$1.2x10^{5}$	$7.5x10^{5}$	$3.4x10^{5}$	$1.5x10^{6}$	**$8.8x10^{5}$**
Vero	Supernatant	1a	Wild-type	42 h	2617.0	1039.0	3039.0	$2.0x10^{5}$	$8.1x10^{4}$	$2.4x10^{5}$	$2.5x10^{6}$	$1.0x10^{6}$	$3.0x10^{6}$	**$2.2x10^{6}$**
Vero	Supernatant	1a	*incA*::GII	42 h	5606.0	4617.0	4028.0	$4.4x10^{5}$	$3.6x10^{5}$	$3.1x10^{5}$	$5.4x10^{6}$	$4.5x10^{6}$	$3.9x10^{6}$	**$4.6x10^{6}$**
Vero	Supernatant	1a	*sinC*::GII	42 h	5450.0	5094.0	5106.0	$4.2x10^{5}$	$4.0x10^{5}$	$4.0x10^{5}$	$5.3x10^{6}$	$4.9x10^{6}$	$5.0x10^{6}$	**$5.1x10^{6}$**
Vero	Supernatant	1a	Wild-type	48 h	3856.3	2523.3	3690.3	$3.0x10^{5}$	$2.0x10^{5}$	$2.9x10^{5}$	$3.7x10^{6}$	$2.5x10^{6}$	$3.6x10^{6}$	**$3.3x10^{6}$**
Vero	Supernatant	1a	*incA*::GII	48 h	4701.3	5101.3	6301.3	$3.7x10^{5}$	$4.0x10^{5}$	$4.9x10^{5}$	$4.6x10^{6}$	$5.0x10^{6}$	$6.1x10^{6}$	**$5.2x10^{6}$**
Vero	Supernatant	1a	*sinC*::GII	48 h	3668.3	2123.3	4645.3	$2.9x10^{5}$	$1.7x10^{5}$	$3.6x10^{5}$	$3.6x10^{6}$	$2.1x10^{6}$	$4.5x10^{6}$	**$3.4x10^{6}$**
Vero	Supernatant	1b	Wild-type	12 h	4.4	3.7	3.3	343.3	285.0	259.4	$4.3x10^{3}$	$3.6x10^{3}$	$3.2x10^{3}$	**$3.7x10^{3}$**
Vero	Supernatant	1b	*incA*::GII	12 h	7.3	4.6	4.9	570.3	354.2	379.8	$7.1x10^{3}$	$4.4x10^{3}$	$4.7x10^{3}$	**$5.4x10^{3}$**
Vero	Supernatant	1b	*sinC*::GII	12 h	3.4	4.0	4.4	267.9	310.7	345.6	$3.3x10^{3}$	$3.9x10^{3}$	$4.3x10^{3}$	**$3.9x10^{3}$**
Vero	Supernatant	1b	Wild-type	24 h	3.2	3.2	1.7	250.8	251.6	129.6	$3.1x10^{3}$	$3.1x10^{3}$	$1.6x10^{3}$	**$2.6x10^{3}$**
Vero	Supernatant	1b	*incA*::GII	24 h	3.3	3.8	3.2	259.4	293.6	250.8	$3.2x10^{3}$	$3.7x10^{3}$	$3.1x10^{3}$	**$3.3x10^{3}$**
Vero	Supernatant	1b	*sinC*::GII	24 h	4.2	3.4	3.7	328.5	267.9	285.0	$4.1x10^{3}$	$3.3x10^{3}$	$3.6x10^{3}$	**$3.7x10^{3}$**
Vero	Supernatant	1b	Wild-type	30 h	78.6	83.9	68.9	$6.1x10^{3}$	$6.5x10^{3}$	$5.4x10^{3}$	$7.6x10^{4}$	$8.2x10^{4}$	$6.7x10^{4}$	**$7.5x10^{4}$**
Vero	Supernatant	1b	*incA*::GII	30 h	162.2	150.8	141.6	$1.3x10^{4}$	$1.2x10^{4}$	$1.1x10^{4}$	$1.6x10^{5}$	$1.5x10^{5}$	$1.4x10^{5}$	**$1.5x10^{5}$**
Vero	Supernatant	1b	*sinC*::GII	30 h	126.1	119.4	122.4	$9.8x10^{3}$	$9.3x10^{3}$	$9.5x10^{3}$	$1.2x10^{5}$	$1.2x10^{5}$	$1.2x10^{5}$	**$1.2x10^{5}$**
Vero	Supernatant	1b	Wild-type	36 h	277.8	366.7	330.0	$2.2x10^{4}$	$2.9x10^{4}$	$2.6x10^{4}$	$2.7x10^{5}$	$3.6x10^{5}$	$3.2x10^{5}$	**$3.2x10^{5}$**
Vero	Supernatant	1b	*incA*::GII	36 h	718.9	757.8	647.8	$5.6x10^{4}$	$5.9x10^{4}$	$5.0x10^{4}$	$7.0x10^{5}$	$7.4x10^{5}$	$6.3x10^{5}$	**$6.9x10^{5}$**
Vero	Supernatant	1b	*sinC*::GII	36 h	403.3	473.3	624.4	$3.1x10^{4}$	$3.7x10^{4}$	$4.9x10^{4}$	$3.9x10^{5}$	$4.6x10^{5}$	$6.1x10^{5}$	**$4.9x10^{5}$**
Vero	Supernatant	1b	Wild-type	42 h	634.4	698.9	801.1	$4.9x10^{4}$	$5.4x10^{4}$	$6.2x10^{4}$	$6.2x10^{5}$	$6.8x10^{5}$	$7.8x10^{5}$	**$6.9x10^{5}$**
Vero	Supernatant	1b	*incA*::GII	42 h	1110.0	828.9	835.5	$8.6x10^{4}$	$6.4x10^{4}$	$6.5x10^{4}$	$1.1x10^{6}$	$8.1x10^{5}$	$8.1x10^{5}$	**$9.0x10^{5}$**
Vero	Supernatant	1b	*sinC*::GII	42 h	936.6	894.4	1007.7	$7.3x10^{4}$	$7.0x10^{4}$	$7.8x10^{4}$	$9.1x10^{5}$	$8.7x10^{5}$	$9.8x10^{5}$	**$9.2x10^{5}$**
Vero	Supernatant	1b	Wild-type	48 h	1085.5	1072.2	1136.6	$8.4x10^{4}$	$8.3x10^{4}$	$8.8x10^{4}$	$1.1x10^{6}$	$1.0x10^{6}$	$1.1x10^{6}$	**$1.1x10^{6}$**
Vero	Supernatant	1b	*incA*::GII	48 h	1680.0	1754.4	1721.1	$1.3x10^{5}$	$1.4x10^{5}$	$1.3x10^{5}$	$1.6x10^{6}$	$1.7x10^{6}$	$1.7x10^{6}$	**$1.7x10^{6}$**
Vero	Supernatant	1b	*sinC*::GII	48 h	1898.9	1872.2	1795.5	$1.5x10^{5}$	$1.5x10^{5}$	$1.4x10^{5}$	$1.8x10^{6}$	$1.8x10^{6}$	$1.7x10^{6}$	**$1.8x10^{6}$**
Vero	Supernatant	2	Wild-type	12 h	6.7	5.6	4.4	517.7	432.2	345.1	$6.5x10^{3}$	$5.4x10^{3}$	$4.3x10^{3}$	**$5.4x10^{3}$**
Vero	Supernatant	2	*incA*::GII	12 h	7.8	7.1	6.3	604.7	555.0	486.6	$7.6x10^{3}$	$6.9x10^{3}$	$6.1x10^{3}$	**$6.9x10^{3}$**
Vero	Supernatant	2	*sinC*::GII	12 h	9.0	6.0	4.9	699.6	466.4	379.3	$8.7x10^{3}$	$5.8x10^{3}$	$4.7x10^{3}$	**$6.4x10^{3}$**
Vero	Supernatant	2	Wild-type	24 h	4.8	5.2	1.8	375.7	402.1	142.5	$4.7x10^{3}$	$5.0x10^{3}$	$1.8x10^{3}$	**$3.8x10^{3}$**
Vero	Supernatant	2	*incA*::GII	24 h	5.3	4.5	6.3	414.6	349.3	487.6	$5.2x10^{3}$	$4.4x10^{3}$	$6.1x10^{3}$	**$5.2x10^{3}$**
Vero	Supernatant	2	*sinC*::GII	24 h	6.3	3.6	5.0	487.6	279.3	385.0	$6.1x10^{3}$	$3.5x10^{3}$	$4.8x10^{3}$	**$4.8x10^{3}$**
Vero	Supernatant	2	Wild-type	30 h	173.8	137.1	113.8	$1.4x10^{4}$	$1.1x10^{4}$	$8.8x10^{3}$	$1.7x10^{5}$	$1.3x10^{5}$	$1.1x10^{5}$	**$1.4x10^{5}$**

Vero	Supernatant	2	*incA*::GII	30 h	517.1	379.4	184.9	4.0x10^{4}	2.9x10^{4}	1.4x10^{4}	5.0x10^{5}	3.7x10^{5}	1.8x10^{5}	**3.5x10^{5}**
Vero	Supernatant	2	*sinC*::GII	30 h	153.8	89.4	50.2	1.2x10^{4}	6.9x10^{3}	3.9x10^{3}	1.5x10^{5}	8.7x10^{4}	4.9x10^{4}	**9.5x10^{4}**
Vero	Supernatant	2	Wild-type	36 h	700.0	989.0	833.0	5.4x10^{4}	7.7x10^{4}	6.5x10^{4}	6.8x10^{5}	9.6x10^{5}	8.1x10^{5}	**8.2x10^{5}**
Vero	Supernatant	2	*incA*::GII	36 h	2133.0	2267.0	989.0	1.7x10^{5}	1.8x10^{5}	7.7x10^{4}	2.1x10^{6}	2.2x10^{6}	9.6x10^{5}	**1.7x10^{6}**
Vero	Supernatant	2	*sinC*::GII	36 h	689.0	344.0	1311.0	5.4x10^{4}	2.7x10^{4}	1.0x10^{5}	6.7x10^{5}	3.3x10^{5}	1.3x10^{6}	**7.6x10^{5}**
Vero	Supernatant	2	Wild-type	42 h	1939.3	816.3	2050.3	1.5x10^{5}	6.3x10^{4}	1.6x10^{5}	1.9x10^{6}	7.9x10^{5}	2.0x10^{6}	**1.6x10^{6}**
Vero	Supernatant	2	*incA*::GII	42 h	3683.3	2972.3	2739.3	2.9x10^{5}	2.3x10^{5}	2.1x10^{5}	3.6x10^{6}	2.9x10^{6}	2.7x10^{6}	**3.0x10^{6}**
Vero	Supernatant	2	*sinC*::GII	42 h	3083.3	2594.3	3261.3	2.4x10^{5}	2.0x10^{5}	2.5x10^{5}	3.0x10^{6}	2.5x10^{6}	3.2x10^{6}	**2.9x10^{6}**
Vero	Supernatant	2	Wild-type	48 h	4041.0	4053.0	4353.0	3.1x10^{5}	3.2x10^{5}	3.4x10^{5}	3.9x10^{6}	3.9x10^{6}	4.2x10^{6}	**4.0x10^{6}**
Vero	Supernatant	2	*incA*::GII	48 h	3886.0	3486.0	4897.0	3.0x10^{5}	2.7x10^{5}	3.8x10^{5}	3.8x10^{6}	3.4x10^{6}	4.8x10^{6}	**4.0x10^{6}**
Vero	Supernatant	2	*sinC*::GII	48 h	3919.0	3886.0	4408.0	3.0x10^{5}	3.0x10^{5}	3.4x10^{5}	3.8x10^{6}	3.8x10^{6}	4.3x10^{6}	**4.0x10^{6}**
Vero	Supernatant	3	Wild-type	12 h	8.9	7.3	6.2	691.3	570.0	483.0	8.6x10^{3}	7.1x10^{3}	6.0x10^{3}	**7.3x10^{3}**
Vero	Supernatant	3	*incA*::GII	12 h	11.8	13.3	11.3	915.2	1036.4	881.0	1.1x10^{4}	1.3x10^{4}	1.1x10^{4}	**1.2x10^{4}**
Vero	Supernatant	3	*sinC*::GII	12 h	7.6	8.2	6.7	587.1	638.4	518.7	7.3x10^{3}	8.0x10^{3}	6.5x10^{3}	**7.3x10^{3}**
Vero	Supernatant	3	Wild-type	24 h	8.5	4.3	3.6	657.1	336.8	279.3	8.2x10^{3}	4.2x10^{3}	3.5x10^{3}	**5.3x10^{3}**
Vero	Supernatant	3	*incA*::GII	24 h	8.0	7.8	9.3	621.3	604.2	725.5	7.8x10^{3}	7.6x10^{3}	9.1x10^{3}	**8.1x10^{3}**
Vero	Supernatant	3	*sinC*::GII	24 h	8.5	5.3	8.0	657.1	414.6	621.3	8.2x10^{3}	5.2x10^{3}	7.8x10^{3}	**7.1x10^{3}**
Vero	Supernatant	3	Wild-type	30 h	281.1	344.5	220.0	2.2x10^{4}	2.7x10^{4}	1.7x10^{4}	2.7x10^{5}	3.3x10^{5}	2.1x10^{5}	**2.7x10^{5}**
Vero	Supernatant	3	*incA*::GII	30 h	734.5	637.8	312.2	5.7x10^{4}	5.0x10^{4}	2.4x10^{4}	7.1x10^{5}	6.2x10^{5}	3.0x10^{5}	**5.5x10^{5}**
Vero	Supernatant	3	*sinC*::GII	30 h	270.0	231.1	336.7	2.1x10^{4}	1.8x10^{4}	2.6x10^{4}	2.6x10^{5}	2.2x10^{5}	3.3x10^{5}	**2.7x10^{5}**
Vero	Supernatant	3	Wild-type	36 h	1211.0	1444.0	956.0	9.4x10^{4}	1.1x10^{5}	7.4x10^{4}	1.2x10^{6}	1.4x10^{6}	9.3x10^{5}	**1.2x10^{6}**
Vero	Supernatant	3	*incA*::GII	36 h	3200.0	3278.0	1578.0	2.5x10^{5}	2.5x10^{5}	1.2x10^{5}	3.1x10^{6}	3.2x10^{6}	1.5x10^{6}	**2.6x10^{6}**
Vero	Supernatant	3	*sinC*::GII	36 h	1289.0	913.0	2278.0	1.0x10^{5}	7.1x10^{4}	1.8x10^{5}	1.3x10^{6}	8.9x10^{5}	2.2x10^{6}	**1.5x10^{6}**
Vero	Supernatant	3	Wild-type	42 h	4799.7	1022.7	4299.7	3.7x10^{5}	7.9x10^{4}	3.3x10^{5}	4.7x10^{6}	9.9x10^{5}	4.2x10^{6}	**3.3x10^{6}**
Vero	Supernatant	3	*incA*::GII	42 h	6833.7	7499.7	6310.7	5.3x10^{5}	5.8x10^{5}	4.9x10^{5}	6.6x10^{6}	7.3x10^{6}	6.1x10^{6}	**6.7x10^{6}**
Vero	Supernatant	3	*sinC*::GII	42 h	8144.7	7055.7	6733.7	6.3x10^{5}	5.5x10^{5}	5.2x10^{5}	7.9x10^{6}	6.9x10^{6}	6.5x10^{6}	**7.1x10^{6}**
Vero	Supernatant	3	Wild-type	48 h	5272.3	5283.3	5772.3	4.1x10^{5}	4.1x10^{5}	4.5x10^{5}	5.1x10^{6}	5.1x10^{6}	5.6x10^{6}	**5.3x10^{6}**
Vero	Supernatant	3	*incA*::GII	48 h	6339.3	6005.3	8039.3	4.9x10^{5}	4.7x10^{5}	6.2x10^{5}	6.2x10^{6}	5.8x10^{6}	7.8x10^{6}	**6.6x10^{6}**
Vero	Supernatant	3	*sinC*::GII	48 h	5328.3	5794.3	6005.3	4.1x10^{5}	4.5x10^{5}	4.7x10^{5}	5.2x10^{6}	5.6x10^{6}	5.8x10^{6}	**5.5x10^{6}**

[a] The number of inclusions/field detected in the optimal dilution was multiplied with the dilution factor. Moreover, the value was corrected for false positives by subtraction of the number of false inclusions detected on average in uninfected wells (< 9 in each experiment).
[b] The number of inclusions/well was calculated by multiplying the number of inclusions/field with the number of fields/well (~77.7).
[c] To calculate the number of IFUs present in the entire collected sample, the number of inclusions/well had to be multiplied by 10, because only a tenth of the sample (20 µl from 200 µl) had been used for the infection. In the case of supernatant samples, the values were also multiplied by 1.25 to account for fact that during supernatant collection only 160 µl (of 200 µl total) supernatant per well were collected and were diluted with 40 µl 5xSPG buffer.
[d] The IFUs present in supernatants collected during the first experiment in Vero cells was quantified twice. Values for both rounds are indicated in the table. Mean values were used in subsequent calculations.
