# Supplementary material for: Insertional mutagenesis in the zoonotic pathogen Chlamydia caviae
Source: PLoS One. 2019 Nov 7;14(11):e0224324. doi: 10.1371/journal.pone.0224324 (PMC6837515; doi:10.1371/journal.pone.0224324)
Supplement: S6 Table — (PDF) [file pone.0224324.s011.pdf]

S6 Table: Calculation of IFUs generated per inclusion for the quantification of infectious progeny. The table depicts a part of the data underlying the graphs displayed in Fig 3A-D (see also S4 and S5 Tables). To calculate the number of IFUs generated per inclusion, the output (number of IFUs in sample) was divided by the input (the number of IFUs in the inoculum used for the infection of each well in the output collection plates).

					Lysate				Supernatant			
						IFUs/inclusion				IFUs/inclusion		
**Cells**	**Exp**	**Strain**	**Time**	**Input (IFUs in inoculum)**	**Output (IFUs in Sample)**	**Individual**	**Mean**	**SD**	**Output (IFUs in Sample)**	**Individual**	**Mean**	**SD**
HeLa	1	Wild-type	12 h	2.2x10^{4}	1.1x10^{3}	0.05			1.0x10^{4}	0.45		
HeLa	2	Wild-type	12 h	1.7x10^{4}	1.1x10^{3}	0.07	**0.09**	0.048	1.1x10^{4}	0.64	**0.57**	0.102
HeLa	3	Wild-type	12 h	1.5x10^{4}	2.2x10^{3}	0.14			9.5x10^{3}	0.62		
HeLa	1	*incA*::GII	12 h	2.8x10^{4}	0.0	0.00			1.2x10^{4}	0.44		
HeLa	2	*incA*::GII	12 h	2.1x10^{4}	0.0	0.00	**0.00**	0.000	1.2x10^{4}	0.59	**0.55**	0.096
HeLa	3	*incA*::GII	12 h	2.5x10^{4}	0.0	0.00			1.5x10^{4}	0.61		
HeLa	1	*sinC*::GII	12 h	2.6x10^{4}	1.1x10^{3}	0.04			8.9x10^{3}	0.34		
HeLa	2	*sinC*::GII	12 h	2.0x10^{4}	2.4x10^{3}	0.12	**0.05**	0.060	9.1x10^{3}	0.45	**0.45**	0.104
HeLa	3	*sinC*::GII	12 h	2.1x10^{4}	0.0	0.00			1.2x10^{4}	0.55		
HeLa	1	Wild-type	24 h	2.2x10^{4}	7.4x10^{3}	0.33			5.6x10^{3}	0.25		
HeLa	2	Wild-type	24 h	1.7x10^{4}	8.1x10^{3}	0.48	**0.39**	0.082	4.4x10^{3}	0.26	**0.37**	0.201
HeLa	3	Wild-type	24 h	1.5x10^{4}	5.3x10^{3}	0.35			9.2x10^{3}	0.60		
HeLa	1	*incA*::GII	24 h	2.8x10^{4}	9.2x10^{3}	0.33			8.3x10^{3}	0.30		
HeLa	2	*incA*::GII	24 h	2.1x10^{4}	1.3x10^{4}	0.63	**0.36**	0.259	4.2x10^{3}	0.20	**0.31**	0.116
HeLa	3	*incA*::GII	24 h	2.5x10^{4}	3.0x10^{3}	0.12			1.1x10^{4}	0.43		
HeLa	1	*sinC*::GII	24 h	2.6x10^{4}	4.9x10^{3}	0.19			4.8x10^{3}	0.18		
HeLa	2	*sinC*::GII	24 h	2.0x10^{4}	2.1x10^{4}	1.04	**0.46**	0.500	3.0x10^{3}	0.15	**0.26**	0.160
HeLa	3	*sinC*::GII	24 h	2.1x10^{4}	3.2x10^{3}	0.15			9.5x10^{3}	0.44		
HeLa	1	Wild-type	30 h	2.2x10^{4}	2.1x10^{6}	96.87			3.0x10^{4}	1.37		
HeLa	2	Wild-type	30 h	1.7x10^{4}	3.2x10^{6}	191.88	**142.08**	47.672	3.0x10^{4}	1.77	**2.08**	0.902
HeLa	3	Wild-type	30 h	1.5x10^{4}	2.1x10^{6}	137.49			4.7x10^{4}	3.09		
HeLa	1	*incA*::GII	30 h	2.8x10^{4}	2.8x10^{6}	100.42			2.0x10^{5}	7.27		
HeLa	2	*incA*::GII	30 h	2.1x10^{4}	3.6x10^{6}	174.54	**127.25**	41.080	1.4x10^{5}	6.81	**7.72**	1.193
HeLa	3	*incA*::GII	30 h	2.5x10^{4}	2.7x10^{6}	106.79			2.3x10^{5}	9.07		
HeLa	1	*sinC*::GII	30 h	2.6x10^{4}	2.7x10^{6}	104.10			8.0x10^{4}	3.09		
HeLa	2	*sinC*::GII	30 h	2.0x10^{4}	3.7x10^{6}	179.86	**136.50**	39.049	6.2x10^{4}	3.05	**3.75**	1.181
HeLa	3	*sinC*::GII	30 h	2.1x10^{4}	2.7x10^{6}	125.55			1.1x10^{5}	5.11		
HeLa	1	Wild-type	36 h	2.2x10^{4}	4.8x10^{6}	219.02			4.9x10^{5}	22.01		
HeLa	2	Wild-type	36 h	1.7x10^{4}	5.5x10^{6}	330.33	**327.8**	107.6	4.9x10^{5}	29.33	**33.22**	13.580
HeLa	3	Wild-type	36 h	1.5x10^{4}	6.6x10^{6}	434.10			7.4x10^{5}	48.32		

HeLa	1	*incA*::GII	36 h	2.8x10^{4}	9.3x10^{6}	334.99			6.1x10^{5}	21.76		
HeLa	2	*incA*::GII	36 h	2.1x10^{4}	7.4x10^{6}	354.02	**368.0**	41.8	4.6x10^{5}	22.11	**25.53**	6.224
HeLa	3	*incA*::GII	36 h	2.5x10^{4}	1.0x10^{7}	415.03			8.3x10^{5}	32.71		
HeLa	1	*sinC*::GII	36 h	2.6x10^{4}	8.9x10^{6}	341.27			2.9x10^{5}	11.10		
HeLa	2	*sinC*::GII	36 h	2.0x10^{4}	6.5x10^{6}	318.63	**412.6**	143.6	1.8x10^{5}	9.04	**12.94**	5.075
HeLa	3	*sinC*::GII	36 h	2.1x10^{4}	1.2x10^{7}	577.90			4.0x10^{5}	18.68		
HeLa	1	Wild-type	42 h	2.2x10^{4}	2.5x10^{7}	1147.13			3.6x10^{6}	163.08		
HeLa	2	Wild-type	42 h	1.7x10^{4}	1.4x10^{7}	844.70	**957.3**	165.3	2.1x10^{6}	127.89	**201.33**	98.310
HeLa	3	Wild-type	42 h	1.5x10^{4}	1.3x10^{7}	880.09			4.8x10^{6}	313.02		
HeLa	1	*incA*::GII	42 h	2.8x10^{4}	2.7x10^{7}	974.20			6.2x10^{6}	221.86		
HeLa	2	*incA*::GII	42 h	2.1x10^{4}	1.6x10^{7}	769.78	**789.7**	175.3	4.1x10^{6}	195.71	**230.65**	40.067
HeLa	3	*incA*::GII	42 h	2.5x10^{4}	1.6x10^{7}	625.22			6.9x10^{6}	274.39		
HeLa	1	*sinC*::GII	42 h	2.6x10^{4}	3.2x10^{7}	1243.70			3.7x10^{6}	141.76		
HeLa	2	*sinC*::GII	42 h	2.0x10^{4}	1.5x10^{7}	732.62	**888.1**	308.7	2.7x10^{6}	130.66	**157.03**	36.492
HeLa	3	*sinC*::GII	42 h	2.1x10^{4}	1.5x10^{7}	688.11			4.3x10^{6}	198.68		
HeLa	1	Wild-type	48 h	2.2x10^{4}	2.0x10^{7}	888.62			4.9x10^{6}	219.37		
HeLa	2	Wild-type	48 h	1.7x10^{4}	1.0x10^{7}	626.34	**809.0**	158.6	3.9x10^{6}	234.59	**305.58**	136.351
HeLa	3	Wild-type	48 h	1.5x10^{4}	1.4x10^{7}	912.04			7.1x10^{6}	462.78		
HeLa	1	*incA*::GII	48 h	2.8x10^{4}	1.3x10^{7}	451.03			6.4x10^{6}	230.73		
HeLa	2	*incA*::GII	48 h	2.1x10^{4}	1.5x10^{7}	723.35	**741.3**	299.6	3.9x10^{6}	187.51	**251.90**	77.189
HeLa	3	*incA*::GII	48 h	2.5x10^{4}	2.6x10^{7}	1049.49			8.5x10^{6}	337.47		
HeLa	1	*sinC*::GII	48 h	2.6x10^{4}	1.3x10^{7}	497.86			3.7x10^{6}	141.45		
HeLa	2	*sinC*::GII	48 h	2.0x10^{4}	1.5x10^{7}	734.79	**758.8**	273.7	2.6x10^{6}	127.03	**180.06**	79.689
HeLa	3	*sinC*::GII	48 h	2.1x10^{4}	2.2x10^{7}	1043.64			5.8x10^{6}	271.70		
Vero	1	Wild-type	12 h	3.8x10^{4}	6.0x10^{3}	0.16			5.4x10^{3}	0.14		
Vero	2	Wild-type	12 h	4.0x10^{4}	0.0	0.00	**0.07**	0.079	5.4x10^{3}	0.13	**0.15**	0.025
Vero	3	Wild-type	12 h	4.0x10^{4}	2.6x10^{3}	0.06			7.3x10^{3}	0.18		
Vero	1	*incA*::GII	12 h	2.7x10^{4}	1.7x10^{3}	0.06			7.8x10^{3}	0.29		
Vero	2	*incA*::GII	12 h	3.3x10^{4}	0.0	0.00	**0.10**	0.125	6.9x10^{3}	0.21	**0.29**	0.077
Vero	3	*incA*::GII	12 h	3.2x10^{4}	7.8x10^{3}	0.24			1.2x10^{4}	0.37		
Vero	1	*sinC*::GII	12 h	3.7x10^{4}	5.2x10^{3}	0.14			4.8x10^{3}	0.13		
Vero	2	*sinC*::GII	12 h	4.1x10^{4}	0.0	0.00	**0.11**	0.098	6.4x10^{3}	0.16	**0.15**	0.023
Vero	3	*sinC*::GII	12 h	4.1x10^{4}	7.8x10^{3}	0.19			7.3x10^{3}	0.18		
Vero	1	Wild-type	24 h	3.8x10^{4}	3.4x10^{4}	0.88			3.6x10^{3}	0.09		
Vero	2	Wild-type	24 h	4.0x10^{4}	0.0	0.00	**0.30**	0.501	3.8x10^{3}	0.10	**0.11**	0.022
Vero	3	Wild-type	24 h	4.0x10^{4}	8.6x10^{2}	0.02			5.3x10^{3}	0.13		
Vero	1	*incA*::GII	24 h	2.7x10^{4}	2.9x10^{4}	1.04			4.4x10^{3}	0.16		
Vero	2	*incA*::GII	24 h	3.3x10^{4}	0.0	0.00	**0.35**	0.603	5.2x10^{3}	0.16	**0.19**	0.052
Vero	3	*incA*::GII	24 h	3.2x10^{4}	0.0	0.00			8.1x10^{3}	0.25		

Vero	1	*sinC*::GII	24 h	$3.7x10^{4}$	$5.7x10^{4}$	1.55			$4.6x10^{3}$	0.13		
Vero	2	*sinC*::GII	24 h	$4.1x10^{4}$	$5.2x10^{3}$	0.13	**0.56**	0.864	$4.8x10^{3}$	0.12	**0.14**	0.029
Vero	3	*sinC*::GII	24 h	$4.1x10^{4}$	0.0	0.00			$7.1x10^{3}$	0.17		
Vero	1	Wild-type	30 h	$3.8x10^{4}$	$5.1x10^{6}$	132.03			$1.5x10^{5}$	4.00		
Vero	2	Wild-type	30 h	$4.0x10^{4}$	$3.9x10^{6}$	96.50	**118.41**	19.159	$1.4x10^{5}$	3.44	**4.75**	1.804
Vero	3	Wild-type	30 h	$4.0x10^{4}$	$5.1x10^{6}$	126.71			$2.7x10^{5}$	6.80		
Vero	1	*incA*::GII	30 h	$2.7x10^{4}$	$5.7x10^{6}$	208.12			$2.9x10^{5}$	10.74		
Vero	2	*incA*::GII	30 h	$3.3x10^{4}$	$3.8x10^{6}$	115.77	**175.73**	51.980	$3.5x10^{5}$	10.78	**12.80**	3.532
Vero	3	*incA*::GII	30 h	$3.2x10^{4}$	$6.6x10^{6}$	203.29			$5.5x10^{5}$	16.87		
Vero	1	*sinC*::GII	30 h	$3.7x10^{4}$	$5.8x10^{6}$	159.09			$1.5x10^{5}$	3.98		
Vero	2	*sinC*::GII	30 h	$4.1x10^{4}$	$4.3x10^{6}$	103.33	**141.37**	32.972	$9.5x10^{4}$	2.31	**4.29**	2.158
Vero	3	*sinC*::GII	30 h	$4.1x10^{4}$	$6.7x10^{6}$	161.70			$2.7x10^{5}$	6.59		
Vero	1	Wild-type	36 h	$3.8x10^{4}$	$1.3x10^{7}$	336.28			$5.6x10^{5}$	14.54		
Vero	2	Wild-type	36 h	$4.0x10^{4}$	$1.0x10^{7}$	254.56	**323.88**	64.018	$8.2x10^{5}$	20.40	**21.33**	7.299
Vero	3	Wild-type	36 h	$4.0x10^{4}$	$1.5x10^{7}$	380.78			$1.2x10^{6}$	29.05		
Vero	1	*incA*::GII	36 h	$2.7x10^{4}$	$1.4x10^{7}$	520.52			$1.5x10^{6}$	56.62		
Vero	2	*incA*::GII	36 h	$3.3x10^{4}$	$1.1x10^{7}$	340.16	**460.10**	103.874	$1.7x10^{6}$	53.70	**63.67**	14.819
Vero	3	*incA*::GII	36 h	$3.2x10^{4}$	$1.7x10^{7}$	519.63			$2.6x10^{6}$	80.70		
Vero	1	*sinC*::GII	36 h	$3.7x10^{4}$	$1.6x10^{7}$	438.94			$6.8x10^{5}$	18.57		
Vero	2	*sinC*::GII	36 h	$4.1x10^{4}$	$1.1x10^{7}$	261.42	**355.91**	89.313	$7.6x10^{5}$	18.43	**24.08**	9.660
Vero	3	*sinC*::GII	36 h	$4.1x10^{4}$	$1.5x10^{7}$	367.36			$1.5x10^{6}$	35.23		
Vero	1	Wild-type	42 h	$3.8x10^{4}$	$1.3x10^{7}$	344.52			$1.4x10^{6}$	37.28		
Vero	2	Wild-type	42 h	$4.0x10^{4}$	$1.3x10^{7}$	313.59	**375.26**	81.504	$1.6x10^{6}$	38.88	**52.53**	25.037
Vero	3	Wild-type	42 h	$4.0x10^{4}$	$1.9x10^{7}$	467.66			$3.3x10^{6}$	81.42		
Vero	1	*incA*::GII	42 h	$2.7x10^{4}$	$1.5x10^{7}$	558.32			$2.8x10^{6}$	101.07		
Vero	2	*incA*::GII	42 h	$3.3x10^{4}$	$1.0x10^{7}$	311.78	**483.58**	149.194	$3.0x10^{6}$	93.62	**133.83**	63.305
Vero	3	*incA*::GII	42 h	$3.2x10^{4}$	$1.9x10^{7}$	580.62			$6.7x10^{6}$	206.80		
Vero	1	*sinC*::GII	42 h	$3.7x10^{4}$	$1.5x10^{7}$	402.05			$3.0x10^{6}$	81.66		
Vero	2	*sinC*::GII	42 h	$4.1x10^{4}$	$1.3x10^{7}$	311.75	**414.33**	109.231	$2.9x10^{6}$	70.30	**108.15**	56.011
Vero	3	*sinC*::GII	42 h	$4.1x10^{4}$	$2.2x10^{7}$	529.18			$7.1x10^{6}$	172.50		
Vero	1	Wild-type	48 h	$3.8x10^{4}$	$9.1x10^{6}$	238.40			$2.2x10^{6}$	56.43		
Vero	2	Wild-type	48 h	$4.0x10^{4}$	$9.2x10^{6}$	230.87	**308.34**	127.720	$4.0x10^{6}$	100.70	**96.16**	37.667
Vero	3	Wild-type	48 h	$4.0x10^{4}$	$1.8x10^{7}$	455.76			$5.3x10^{6}$	131.35		
Vero	1	*incA*::GII	48 h	$2.7x10^{4}$	$1.4x10^{7}$	530.40			$3.4x10^{6}$	126.21		
Vero	2	*incA*::GII	48 h	$3.3x10^{4}$	$8.6x10^{6}$	263.07	**494.47**	215.686	$4.0x10^{6}$	122.26	**150.89**	46.210
Vero	3	*incA*::GII	48 h	$3.2x10^{4}$	$2.2x10^{7}$	689.93			$6.6x10^{6}$	204.20		
Vero	1	*sinC*::GII	48 h	$3.7x10^{4}$	$1.3x10^{7}$	346.78			$2.6x10^{6}$	70.69		
Vero	2	*sinC*::GII	48 h	$4.1x10^{4}$	$1.1x10^{7}$	265.01	**370.40**	118.978	$4.0x10^{6}$	96.05	**100.48**	32.236
Vero	3	*sinC*::GII	48 h	$4.1x10^{4}$	$2.1x10^{7}$	499.42			$5.5x10^{6}$	134.70		
