# Supplementary material for: Insertional mutagenesis in the zoonotic pathogen Chlamydia caviae
Source: PLoS One. 2019 Nov 7;14(11):e0224324. doi: 10.1371/journal.pone.0224324 (PMC6837515; doi:10.1371/journal.pone.0224324)
Supplement: S7 Table — (PDF) [file pone.0224324.s012.pdf]

Table S7: Quantitative assessment of host cell lysis at late infection stages. The table depicts the raw data underlying the graph displayed in Fig 3E. Shown are blank-subtracted absorbance values from the LDH activity assay, as well as the percentage of dead cells (obtained after normalization to the total cell lysate).

	Condition	Time point	Exp1	Exp2	Exp3	Mean	SD
**Blank-subtracted absorbance values** (sample$_{(Abs\ 490\text{-}690)}$ – blank$_{(Abs\ 490\text{-}690)}$)	Uninfected	24 hpi	0.021	0.019	0.021	0.020	0.001
	Wild-type	24 hpi	0.016	0.005	0.007	0.010	0.006
	incA::GII	24 hpi	0.015	0.013	0.024	0.017	0.006
	sinC::GII	24 hpi	0.008	0.011	0.020	0.013	0.006
	Total lysate	24 hpi	0.326	0.325	0.351	0.334	0.014
	Uninfected	30 hpi	0.021	0.019	0.022	0.021	0.001
	Wild-type	30 hpi	0.016	0.019	0.087	0.041	0.040
	incA::GII	30 hpi	0.015	0.037	0.087	0.047	0.037
	sinC::GII	30 hpi	0.071	0.030	0.072	0.058	0.024
	Total lysate	30 hpi	0.282	0.282	0.300	0.288	0.011
	Uninfected	36 hpi	0.023	0.027	0.027	0.026	0.003
	Wild-type	36 hpi	0.065	0.053	0.104	0.074	0.027
	incA::GII	36 hpi	0.063	0.066	0.150	0.093	0.050
	sinC::GII	36 hpi	0.096	0.033	0.096	0.075	0.037
	Total lysate	36 hpi	0.303	0.339	0.327	0.323	0.019
	Uninfected	40 hpi	0.024	0.025	0.034	0.027	0.006
	Wild-type	40 hpi	0.147	0.091	0.161	0.133	0.037
	incA::GII	40 hpi	0.127	0.127	0.164	0.140	0.021
	sinC::GII	40 hpi	0.090	0.196	0.117	0.134	0.055
	Total lysate	40 hpi	0.271	0.296	0.297	0.288	0.015
	Uninfected	48 hpi	0.029	0.029	0.034	0.030	0.003
	Wild-type	48 hpi	0.274	0.212	0.282	0.256	0.039
	incA::GII	48 hpi	0.356	0.301	0.287	0.314	0.037
	sinC::GII	48 hpi	0.216	0.309	0.231	0.252	0.050
	Total lysate	48 hpi	0.298	0.368	0.336	0.334	0.035
**Percentage of dead cells** (sample/total lysate)*100	Uninfected	24 hpi	6.4	5.9	6.1	6.1	0.234
	Wild-type	24 hpi	5.0	1.7	2.0	2.9	1.806
	incA::GII	24 hpi	4.6	3.9	6.9	5.1	1.584
	sinC::GII	24 hpi	2.5	3.3	5.7	3.8	1.652
	Total lysate	24 hpi	100.0	100.0	100.0	100.0	0.000
	Uninfected	30 hpi	7.3	6.8	7.3	7.1	0.302
	Wild-type	30 hpi	5.6	6.8	28.9	13.8	13.115
	incA::GII	30 hpi	5.4	13.2	29.1	15.9	12.089
	sinC::GII	30 hpi	25.1	10.7	24.0	19.9	7.999
	Total lysate	30 hpi	100.0	100.0	100.0	100.0	0.000
	Uninfected	36 hpi	7.5	8.0	8.4	8.0	0.411
	Wild-type	36 hpi	21.3	15.7	31.8	22.9	8.194
	incA::GII	36 hpi	21.0	19.4	46.0	28.8	14.943
	sinC::GII	36 hpi	31.9	9.7	29.4	23.7	12.185
	Total lysate	36 hpi	100.0	100.0	100.0	100.0	0.000
	Uninfected	40 hpi	8.7	8.5	11.4	9.5	1.621
	Wild-type	40 hpi	54.3	30.9	54.2	46.5	13.482
	incA::GII	40 hpi	47.0	43.1	55.4	48.5	6.315
	sinC::GII	40 hpi	33.4	66.3	39.3	46.3	17.526
	Total lysate	40 hpi	100.0	100.0	100.0	100.0	0.000
	Uninfected	48 hpi	9.6	7.8	10.2	9.2	1.268
	Wild-type	48 hpi	92.0	57.4	84.0	77.8	18.107
	incA::GII	48 hpi	119.4	81.7	85.3	95.5	20.800
	sinC::GII	48 hpi	72.4	83.8	68.9	75.0	7.790
	Total lysate	48 hpi	100.0	100.0	100.0	100.0	0.000
