# Supplementary material for: Insertional mutagenesis in the zoonotic pathogen Chlamydia caviae
Source: PLoS One. 2019 Nov 7;14(11):e0224324. doi: 10.1371/journal.pone.0224324 (PMC6837515; doi:10.1371/journal.pone.0224324)
Supplement: S8 Table — (PDF) [file pone.0224324.s013.pdf]

S8 Table: Monitoring of chicken embryo death and survival. The table depicts the raw data underlying the graph displayed in Fig 3F. Displayed are observed time points of chicken embryo death (in hours post injection; S.U.E: survived until endpoint; I.F: injection failure, egg excluded at the beginning of the experiment) and the calculated proportions of surviving embryos over the course of time.

Recorded chicken embryo deaths [hours post injection]				
**Exp1**	**Wild-type**	***sinC*::GII**	***incA*::GII**	**Mock**
Egg 1	113	125	121	S.U.E
Egg 2	119	131	125	S.U.E
Egg 3	125	133	129	S.U.E
Egg 4	127	137	129	S.U.E
Egg 5	I.F	137	I.F	S.U.E
**Exp2**	**Wild-type**	***sinC*::GII**	***incA*::GII**	**Mock**
Egg 1	117	125	119	S.U.E
Egg 2	117	125	119	S.U.E
Egg 3	123	132	119	S.U.E
Egg 4	123	135	123	S.U.E
Egg 5	129	137	123	S.U.E
Egg 6	132	139	125	S.U.E
Egg 7	139	169	I.F	S.U.E
**Exp3**	**Wild-type**	***sinC*::GII**	***incA*::GII**	**Mock**
Egg 1	70	119	115	S.U.E
Egg 2	113	127	117	S.U.E
Egg 3	117	130	117	S.U.E
Egg 4	121	133	119	S.U.E
Egg 5	130	133	121	S.U.E
Egg 6	130	137	130	S.U.E
Egg 7	143	137	133	S.U.E
**Total n**	**18**	**19**	**17**	**19**

Survival [hours post injection]				
**Time [hpi]**	**Wild-type**	***sinC*::GII**	***incA*::GII**	**Mock**
0	100.0	100.0	100.0	100.0
70	94.4	100.0	100.0	100.0
113	83.3	100.0	100.0	100.0
115	83.3	100.0	94.1	100.0
117	66.7	100.0	82.4	100.0
119	61.1	94.7	58.8	100.0
121	55.6	94.7	47.1	100.0
123	44.4	94.7	35.3	100.0
125	38.9	78.9	23.5	100.0
127	33.3	73.7	23.5	100.0
129	27.8	73.7	11.8	100.0
130	16.7	68.4	5.9	100.0
131	16.7	63.2	5.9	100.0
132	11.1	57.9	5.9	100.0
133	11.1	42.1	0.0	100.0
135	11.1	36.8	0.0	100.0
137	11.1	10.5	0.0	100.0
139	5.6	5.3	0.0	100.0
